# Nondestructive tracking of viral infections by viral protein-initiated fluorescent sensors

**DOI:** 10.1128/jvi.00044-26

**Published:** 2026-03-17

**Authors:** Zuoxi Zhang, Xianhuang Li, Kexin Yin, Chunguang Yin, Bowen Li, Wenchang Peng, Jinsong Han, Boyu Luo, Yue Teng, Sheng Xie, Tao Wang, Bin Zheng

**Affiliations:** 1 Faculty of Medicine, Tianjin University 12605, Tianjin, China; 2 The Province and Ministry Co-sponsored Collaborative Innovation Center for Medical Epigenetics, School of Biomedical Engineering and Technology, Tianjin Medical University 12610, Tianjin, China; 3 State Key Laboratory of Chemo and Biosensing, College of Chemistry, Hunan University 214172 , Changsha, China; 4 State Key Laboratory of Natural Medicines, National R&D Center for Chinese Herbal Medicine Processing, Department of Food Quality and Safety, College of Engineering, China Pharmaceutical University 429365 , Nanjing, China; 5 State Key Laboratory of Pathogen and Biosecurity Beijing Institute of Microbiology and Epidemiology, Academy of Military Medical Sciences 602528 , Beijing, China; 6 Shenzhen Research Institute of Hunan University, Hunan University, Shenzhen, China; St Jude Children's Research Hospital, Memphis, Tennessee, USA

**Keywords:** viral protein-based enhanced targeting, molecularly induced targeting buffer effect, viral particle tracking

## Abstract

Real-time tracking of viral infection processes is critical for elucidating the pathogenic mechanisms and therapeutic targets. A more detailed understanding of viral movement in the extracellular environment *in vivo* as well as in extraplasmic space requires the development of a tool capable of stable fluorescence without affecting the virus’s own physiological chemotaxis. Herein, we report a viral protein-based enhanced targeting (PBET) fluorescent sensor that can image the infection process of live viruses in spaces adjacent to living cells and *in vivo* environments. Experimental and computational studies have shown that PBET sensors are capable of spontaneous dimer formation and have an enhanced ability to target the electrostatic interactions of functionalized motifs through the restriction of intramolecular motions. The PBET sensor effectively ensured that the attachment process did not affect the physicochemical properties of the virus because of the molecularly induced targeting buffer effect (MITB) binding to the viral surface proteins. Meanwhile, fluorescence intensity, persistence, and minimum labeling concentration, all several times higher than existing fluorescent dyes, ensure that this sensor element can stably and efficiently track viral particle infection timelines at the cellular and tissue levels. This provides a novel platform strategy for antiviral localization studies and the diagnosis of infected areas in patients.

**IMPORTANCE:**

Real-time viral tracer technology plays a driving role in deconstructing viral infections by demonstrating the infection process and the potential binding regions in multiple dimensions. However, the methods of labeling viruses are still limited to traditional fluorescent protein labeling and loaded fluorescent molecules. These schemes have undoubtedly had a noticeable impact on simple viral structures, with labeled viruses showing fluctuations in infectivity. Therefore, it is important to nondestructively perform the dynamic tracing of viruses. By integrating the existing protein fluorescent labeling schemes and fluorescent molecular charge transfer theory, this study builds a system with nondestructive labeling, dynamic tracing, and labeling mechanism analysis of viruses, which is of great scientific value in the prevention of infectious diseases.

## INTRODUCTION

Visualization techniques for tracking viral dynamics in extracellular spaces have been shown to detect viral infection cycles and the recognition process when viruses are exposed to cells (1–6). This technology provides both static and dynamic imaging information for the development of antiviral drugs and exploration of clinical strategies. However, owing to limitations in the molecular weight and spatial volume of fluorescent materials, nondestructive modification for virus tracing remains unachievable (7–11). Existing tracing methods employ covalent linkages to label viral proteins or nucleic acids with fluorescent molecules (12–14). For instance, quantum dot (QD) labeling has emerged as a novel viral tracing approach that enables fluorescent imaging analysis. Through the process of intra-viral assembly, QDs are encapsulated within viral particles, thereby facilitating the tracking of viruses in extracellular environments and during nucleic acid injection (2, 15–20). Furthermore, advances in protein binding and labeling techniques have enabled the application of numerous fluorescent molecules that covalently bind to protein residues for pathogen tracing (21–28). These molecules have the capacity to bind to lysine or cysteine residues on proteins, thereby forming complexes between the fluorescent label and pathogen protein. However, the size of viral particles and the high cellular affinity of viral protein amino acid residues pose challenges to the feasibility of fluorescent labeling and tracking. On one hand, synthesizing fluorescent proteins and nanoparticles with small particle sizes is challenging, and existing fluorescent particles ranging from 50 to 200 nm significantly affect viral protein assembly and target protein affinity during infection (29–33). Consequently, the development of strategies for labeling proteins with small molecules would facilitate more direct visualization approaches for the analysis of viral infection processes and the identification of viral target proteins.

Viral proteins, which serve as primary structural components of viral particles and anchor functional units for infection, have become crucial targets for viral analysis. Furthermore, the structural conformation of viral proteins has been demonstrated to significantly influence viral subtype infectivity and survival capacity (34–40). Despite significant variations in protein composition, amino acid sequences, and structures among different viruses, convergent evolution has established a similar cellular infection pathway for these morphologically diverse viruses, relying on the charge properties of the viral surface proteins (41–44). During evolution, viruses have developed the capacity to recognize and evade complex biological environments. The key to this development is the presence of positively charged pockets on the surface protein interface (45–50). It has been established that the isoelectric points of viral proteins are not significantly different from those of cellular proteins. In both cases, a negative charge was observed at physiological pH. However, viruses possess distinct positively charged pockets, formed by pockets of positively charged amino acid side chains. This surface charge facilitates electrostatic adhesion between viral particles and cellular proteins, particularly membrane proteins, thereby enabling infection of target organs. Conversely, these positively charged pockets form protein caps during viral entry, enabling camouflage to evade recognition (51–56). Consequently, the identification of binding pockets through the analysis of differences in surface charge distribution and amino acid sequence composition facilitates tight binding to viral proteins and specific viral attachment during tracing (57). The development of the viral sensor capable of electrostatic binding to form fluorescently labeled complexes with viruses is of significant research value and has considerable potential for application.

Positively charged chemotactic groups, defined as a class of targeting groups capable of achieving positive charge attraction, can be engineered for electrostatic binding to viral proteins. Existing tools that utilize positively charged chemotactic groups for protein targeting have a range of applications, including anti-viral, anti-bacterial, and anti-tumor therapies. In particular, the electronic properties of nitrogen atoms enable the incorporation of nitrogen-containing heterocycles into organic structures. These heterocycles not only function as positively charged chemotactic groups but also facilitate intramolecular charge transfer. Furthermore, nitrogen-rich compounds containing azide, triazole, tetraazole, and quinoline groups have been extensively utilized in biological protein-labeling systems (58–60). The utilization of nitrogen-containing heterocyclic molecules to interact with proteins and label viral surface proteins with fluorescent molecules within minutes, non-quantifiable volumes offers novel approaches and alternative solutions for viral tracking (61, 62). Another critical factor in electrostatic adsorption labeling by positively charged chemotactic groups is selection of the fluorescent core. The fluorescent core is characterized by exceptional emission intensity, which is attributable to intramolecular interactions and electron orbital distribution. It is evident that within the context of diverse charge-induced luminescence mechanisms in fluorescent elements, the inhibition of molecular motion through aggregation to facilitate the release of fluorescence results in the occurrence of hyper-luminescence within charge-accumulating pockets of target proteins (63–65).

**Fig 1:**
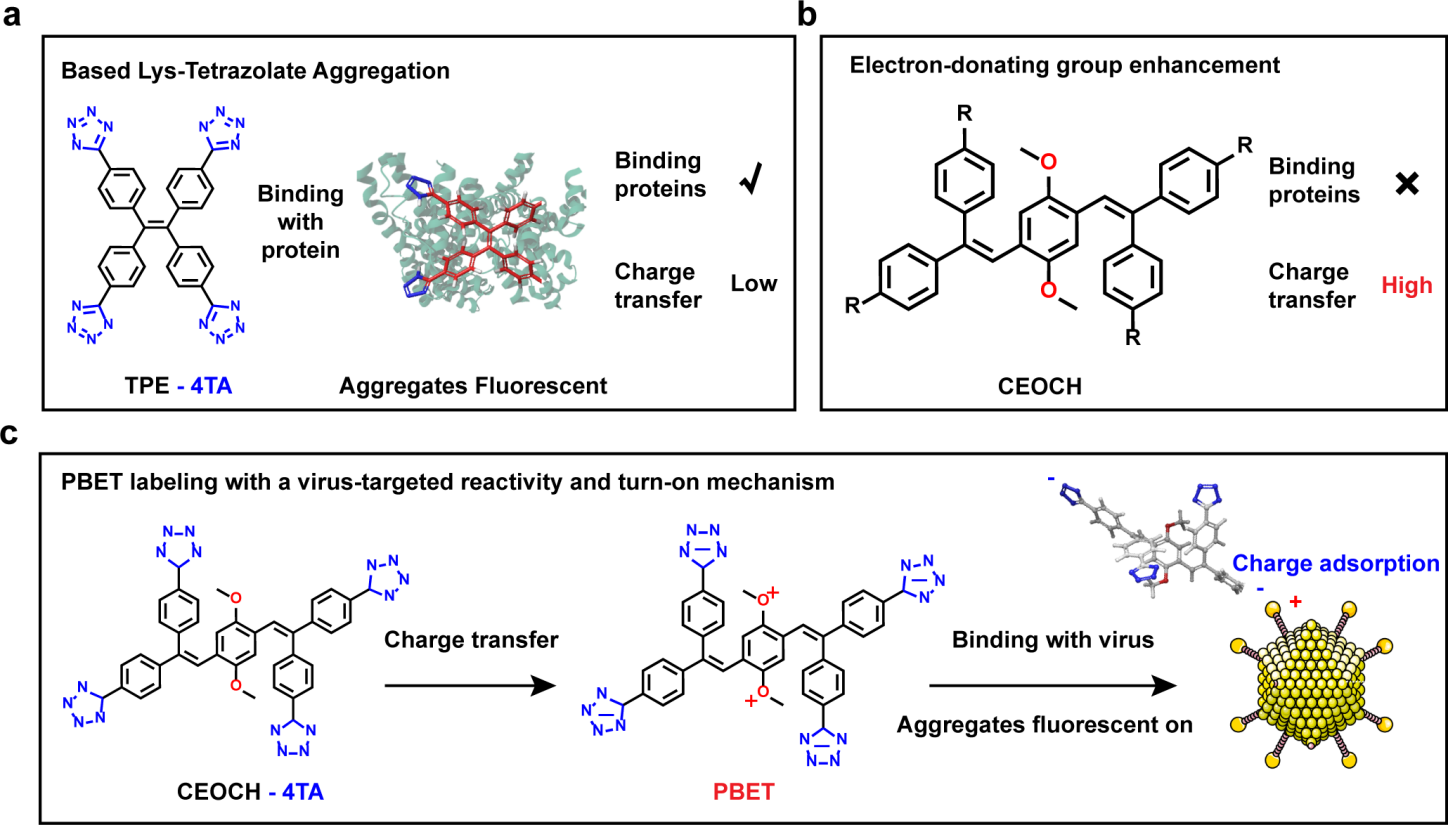
Previously reported protein sensor and PBET-sensing concepts. (**a**) Sensing mechanism of the existing TPE-4TA. (**b**) Hypothetical fluorescent probes that do not aggregate in response to viral surface proteins. (**c**) Sensing concept using a PBET sensor (this work). Rn, Positively charged chemotactic group.

The present strategy is an advancement of the existing charge-adsorption labeling approaches for proteins (1,1,2,2-tetrakis(4-(1H-tetrazol-5-yl)phenyl)ethene (TPE-4TA)). Nitrogen-containing heterocyclic compounds are utilized to address complex *in vivo* environments via electron transfer (Fig. 1a) (66–68). The tetrazole (TA) molecule was selected as an excellent charge-transfer unit because its electron-withdrawing nitrogen atoms confer strong positively charged chemotactic properties to the tetrazole group within the solvent (69). The positively charged chemotactic group selection process required a fluorescent core that was capable of supporting and facilitating electron transfer. Screening of fluorescent molecular libraries led to the identification of a fluorescent core that exhibited a high degree of twisting, with the capacity to form intramolecular hydrogen bonds: ((2,5-dimethoxy-1,4-phenyl)bis(ethylene-2,1,1-triyl))-tetraphenyl (CEOCH) (Fig. 1b) (70, 71). The tetraazole-modified CEOCH fluorescent sensor, which was designed and constructed for this purpose, leveraged the positively charged chemotactic properties of the tetraazole group. This property enables it to bind to positively charged aggregation pockets within viral proteins and rapidly aggregate. Concurrently, the resultant polymeric compound augments protein-binding stability, thereby forming a fluorescently labeled complex with the viral particle. This fluorescence sensor, based on protein-based enhanced targeting (PBET), was systematically named the PBET sensor (Fig. 1c). Furthermore, the electron transfer properties of the fluorescence sensor molecule were leveraged to form a charge buffer layer on the outer surface of the viral protein, thereby protecting the surface potential of viral proteins. This phenomenon is referred to as molecular-induced targeted buffering (MITB). It has been demonstrated that this property can rectify surface charge alterations induced by the electrostatic adsorption of conventional markers. Furthermore, it has been shown to restore protein interactions during the course of infection, thereby ensuring the preservation of viral physiological activity. Specifically, electrostatic adsorption between positively charged pockets on viral proteins and positively charged chemotactic group-modified fluorescent cores was used to design a class of virus protein-targeted enhanced fluorescence sensors. The sensor has been shown to bind tightly to positively charged pockets on viral capsids and envelopes via electrostatic adsorption feedback enhancement, thereby enabling stable and nondestructive tracking of the spatial localization of live viruses during infection. This highly efficient, high-binding-energy, stable, and biocompatible nondestructive binding strategy offers a promising and versatile approach for live virus-related research, such as the real-time tracking of unknown wild-type viruses or highly pathogenic mutants.

## MATERIALS AND METHODS

### General information

The adenovirus serotype 5 (AD5) strain was a kind gift from Prof. Zhuozhuang Lu’s group at the Chinese Center for Disease Control and Prevention. The Sendai virus (SEV) BB1 strain was a kind gift from Dr. Lishu Zheng’s group at the Chinese Center for Disease Control and Prevention. The enterovirus D68 strain (EV-D68; GenBank accession number KU844179.1) was a kind gift from Prof. Xiaofang Yu’s group at Johns Hopkins University. Influenza A/WSN (WSN) preserved by Tao Wang’s group at Tianjin University. Human embryonic kidney 293A (293A) cells, rhabdomyosarcoma (RD) cells, and Madin-Darby canine kidney (MDCK) cells were cultured in high-glucose Dulbecco’s modified Eagle’s medium (DMEM; Invitrogen), supplemented with 10% fetal bovine serum (FBS; Ausbian) and 1% penicillin/streptomycin (Invitrogen) at 37°C and 5% CO2. Healthy female BALB/C nude mice were purchased from HFK Technology Co., Ltd. (Beijing).

### Sensor preparation

These fluorescent dyes were either developed in our laboratory or donated by companies. Many of the tetrazole-functionalized sensors were newly designed and prepared via organic synthesis. See Fig. S3 for the preparation process of the PBET sensor.

### Preparation of the virus for labeling

EV-D68 was prepared in RD cell culture as previously reported. AD5 was prepared in 293A cell culture by incubation with the viral suspension, as previously reported. Once the cells displayed 80% cytopathic effects, the cell culture medium was subjected to three freeze-thaw cycles, followed by centrifugation at 2000 × *g* for 15 min at 4°C to remove cellular debris. The culture supernatants containing viruses were carefully harvested by ultracentrifugation through SDGC at 110,000 × *g* for 2 h in an SW41 rotor (Beckman Coulter Inc., Germany). After sucrose was removed, the viruses were sub-packaged and stored at −80°C. SEV viruses were propagated in 10-day-old embryonated eggs for 48 h at 37°C, as previously reported. Subsequently, viruses in allantoic fluid were harvested and purified by SDGC at 110,000 × *g* in an SW 41 rotor for 2 h at 4°C.

### Virus labeling protocol

First, pre-treat the dye molecules by dissolving the PBET sensor in deionized distilled water at room temperature and sonicating it in a water bath for 5 min. Immediately centrifuge at 1,000 × *g* and collect the supernatant. Next, place pre-chilled purified virus (or an equivalent amount of viral protein) dispersed in various fresh media (e.g., DMEM) into a 10 kDa ultrafiltration tube. Centrifuge at 10,000 × *g* for 5 min at 4°C, then redissolve in DMEM to remove residual cellular components from the virus purification process. Mix the obtained PBET solution at a specific concentration with the viral particle solution for labeling. Specifically, mix the working-concentration PBET concentrate with the viral solution at a 20:1 virus/PBET ratio (e.g., add 50 μL PBET solution to 1 mL viral solution). Stir at 4°C in the dark for 30–60 min (extending reaction time does not enhance viral labeling brightness). Transfer the labeled solution to an ultrafiltration tube (10 kDa, Millipore). Centrifuge at 10,000 × *g* for 5 min at 4°C. Resuspend the fluorescently labeled complex in fresh sterile DMEM. Repeat washing three times to remove unlabeled and loosely bound PBET, yielding a stable solution of fluorescently labeled complexes with firmly bound PBET. Typically, aliquot the separated solution to avoid freeze-thaw effects on the virus for subsequent use.

### Modeling of molecular docking

The PBET sensor or complexes, the VP protein (PDB code: 4WM7) in EV-D68, capsid protein VP1 (PDB code: 7KFR) in adeno-associated virus, and virus F protein in the post-fusion conformation (PDB code: 3MAW) were pre-processed separately via AutoDock Tools 1.6 to generate PDBQT files for molecular docking; PBET and the protein were docked using Vina 1.1.2, and the conformations with the lowest binding energies were selected for analysis. The interactions between proteins and PBET were analyzed using Plip 2021, and three-dimensional interaction maps were drawn using PyMOL 3.0.

### Real-time qPCR

Total RNA was extracted using Trizol (Qiagen). RNAs were reverse-transcribed to cDNA via the TransScript First-Strand cDNA Synthesis SuperMix Kit in accordance with the manufacturer’s instructions. Quantitative real-time PCR (RT-qPCR) was performed using TransStart Top Green q-PCR SuperMix (TransGen, China) in a 20 µL reaction volume (10 µL TransStart Top Green q-PCR SuperMix, 200 mM forward and reverse primers, 2 µL cDNA template). The following cycling conditions were used: 94°C for 30 s and 40 cycles of 94°C for 5 s and 60°C for 30 s. The reaction was run on a LightCycler 96 Real-Time PCR machine (Roche), and the levels of gene mRNAs were normalized to those of GAPDH mRNA.

### TCID_50_ assay

The infectivity of TPE-labeled viruses was quantified by the 50% tissue culture infective dose (TCID_50_). For the TCID_50_ assay, cells (RD, 293A, and MDCK) were cultured in 96-well plates in culture medium until they reached 80%–90% confluence. The labeled and nonlabeled viruses were diluted 10^n^-fold, ranging from 10^−4^ to 10^−9^ in infection culture media.

### Statistical analyses

The data were shown as mean value ± the standard deviation of independent experiments. *In vivo* mouse experiments were set as *n* ≥ 3 for each group. One-way ANOVA and Student’s *t*-test were utilized for statistical analysis. Values of *P ≤ 0.05 and **P ≤ 0.01 were applied to annotate statistical significance.

## RESULTS

### Efficient viral sensor based on a CEOCH fluorescent core

We first screened the molecular cores in the fluorescent library to identify those that have the potential to be linked to viruses via electrostatic adsorption and whose fluorescence intensity can still be observed with excellent detectability properties. Previous studies have shown that previous materials can be negatively charged in the free state through the introduction of nitrogen-containing heterocycles and can electrostatically adsorb to amino acid residues with a positive charge (e.g., Lys) on the surface of proteins. During this binding process, the bound hydrophobic sites restrict molecular motion, allowing fluorescence. However, such molecular structures typically exist as aggregated fluorescent particles in aqueous solutions due to their self-assembly properties. The presence of these fluorescent aggregates not only reduces the molecules’ water solubility but also increases background noise and false positives during viral labeling. Therefore, we first evaluated those fluorescent probes with good water solubility from various fluorescent probe libraries and screened those fluorescent cores that are non-fluorescent or weakly fluorescent in a single-molecule aqueous solution (Fig. S1a). It was found that CEOCH could be an excellent fluorescent core with both a strong chemical structure charge transfer ability and a high signal-to-noise ratio of the fluorescence signal. Fluorescence analysis indicates that the electron transfer properties of CEOCH confer the potential for positive charge chemotaxis via the TA group while ensuring the stability of the fluorescent core (Fig. S1a and b).

**Fig 2:**
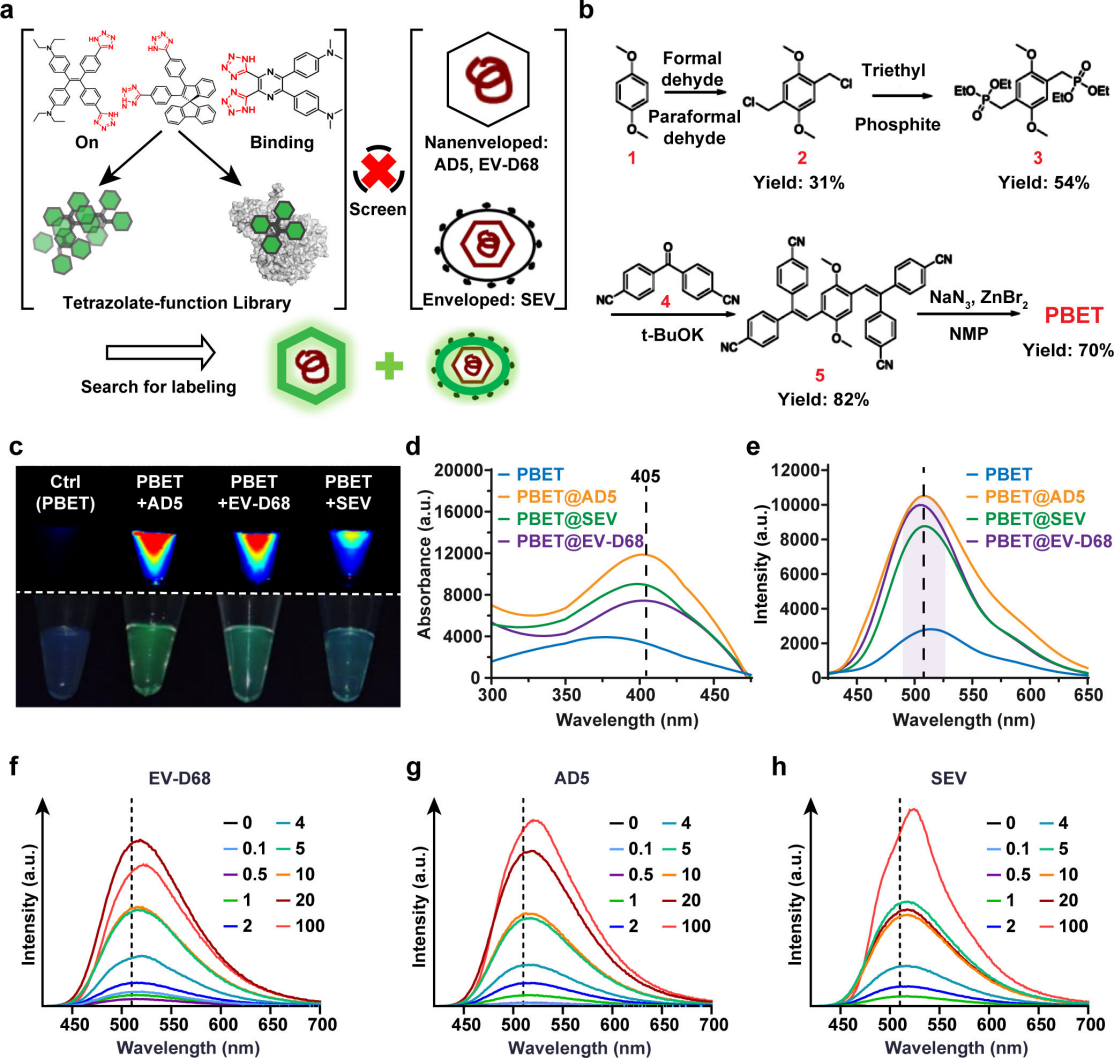
Sensor synthesis and screening strategy for labeled viruses. (**a**) Schematic of the principle of screening fluorescent libraries for fluorescent virus labeling. (**b**) PBET sensor synthesis pathway. (**c**) Fluorescence images of PBET solutions and mixtures with different strains of viruses. (**d**) Corresponding fluorescence excitation spectra and (**e**) emission spectra of samples of PBET mixed with viruses. (**f**) Fluorescence spectra of PBET@EV-D68 fluorescently labeled complexes formed with EV-D68 virus at different PBET concentrations in DMEM. (**g**) Fluorescence spectra of PBET@AD5 fluorescently labeled complexes formed with AD5 virus at different PBET concentrations in DMEM. (**h**) Fluorescence spectra of PBET@SEV fluorescently labeled complexes formed with SEV virus at different PBET concentrations in DMEM. [Virus] = 10^7^ TCID50 mL^−1^.

We subsequently tested whether CEOCH, which has undergone side-chain heterocyclization, would produce a fluorescent response upon binding to the virus. For this purpose, we prepared a variety of side chain derivatives, including affinity functional groups such as azides. Fluorescence intensity tests revealed that the sensor with the nitrogen heterocycle as the side chain could react strongly with viruses. In contrast, the five-membered carbocyclic side chain was completely insensitive (Fig. S1a). By changing the targeting moiety for secondary screening, we tested the response sensitivity in a physiological chemotaxis environment using envelopment-free virus (AD5) (72) and enveloped virus (SEV) as model targets. In our experiments, we simulated different environments with water, DMEM, and culture broth to compare and track the changes in fluorescence intensity before and after mixing with viruses (Fig. S1a). The tetrazolium moiety exhibited excellent reactivity differences, and this binding did not affect the fluorescence ability of the CEOCH core. Moreover, we synthesized TPE-4TA for codetection by modifying the TPE core and found that the tetrazole-functionalized TPE fluorescent sensor could exhibit weak fluorescence when mixed with a virus mixture, but these labeled viruses were not bright enough under fluorescence microscopy, resulting in the detection of such molecules with a low signal‒to‒noise ratio (Fig. 2a; Fig. S1b). To achieve stable virus tracking, we optimized the synthetic pathway and finally synthesized stable PBET molecules (Fig. 2b; Fig. S2 to S4).

### The PBET sensor is a robust viral sensor that combines viral targeting and affinity

Fascinatingly, PBET demonstrates properties at the photophysical level, including spectral characteristics, brightness, and dynamic range, and this excellence is attributed to the molecular motion of PBET, which enhances intramolecular charge transfer, which in turn permits the sensor to efficiently target the viral surface, which limits molecular motion when attached connections are made and thus releases intense fluorescence. To further validate its reactivity and labeling ability, we fluorescently labeled a variety of viruses via PBET to characterize their fluorescence intensity. In the experiment, an equal amount of wild-type natural virus was added to a solution of PBET, and strong blue‒green fluorescence was detected (Fig. 2c). The fluorescence turn-on effect in the broad blue-green spectrum was clearly shown under the imaging system (Fig. 2d). For three different virus strains, the fluorescence emission of the mixture peaked at approximately 510 nm (Fig. 2e). Conversely, the virus-free dye solution showed weak luminescence at 510 nm. The fluorescence intensity at 510 nm was enhanced more than 6-fold. In order to ascertain the optimal working concentration of PBET for viral labeling, a combination of viral particles and PBET concentration gradients was utilized, and fluorescence spectroscopy scans were performed on the labeled complexes. The results demonstrated that there were no statistically significant differences in the PBET concentration-response curves between enveloped and non-enveloped viruses. Furthermore, excellent fluorescence intensity per unit concentration was observed at 5 μM (Fig. 2f through h; Fig. S5a through f).

**Fig 3:**
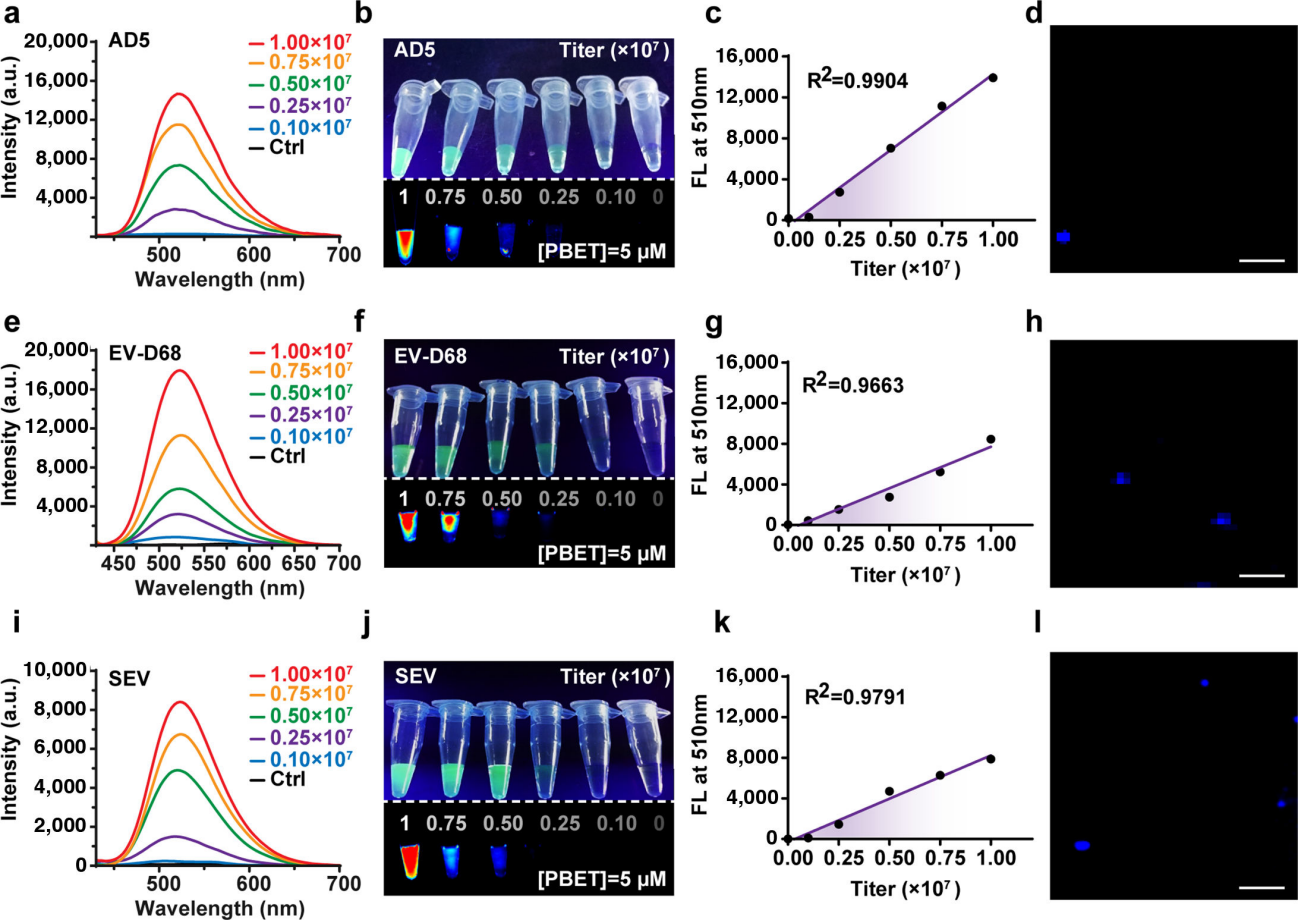
Characterization of the fluorescent labeling of non-enveloped and enveloped viruses. (**a**) Fluorescence emission spectra of solutions of different titers of the non-enveloped virus AD5; (**e**) non-enveloped virus EV-D68; and (**i**) enveloped virus SEV mixed with PBET, respectively. (**b**) Fluorescence images of PBET@AD5 solution under 405 nm excitation light; (**f**) PBET@EV-D68; and (**J**) PBET@SEV, obtained via a 488 nm high-pass filter. (**c**) PBET with different titers of non-enveloped virus AD5; (**g**) non-enveloped virus EV-D68; and (**k**) fluorescence intensity at 510 nm released by mixing the enveloped virus SEV with the corresponding viral particle titers. (**d**) Non-enveloped virus AD5; (**h**) non-enveloped virus EV-D68; and (**l**) viral particle labeling fluorescence images obtained by confocal microscopy of enveloped virus SEV after mixing and labeling with PBET for 2 h. The blue dots in the figure represent viruses after fluorescent labeling. [PBET] = 5 µM; scale bar: 10 µm.

Furthermore, fluorescence titration studies revealed that when the virus titer increased, the fluorescence intensity increased in a quasi-linear manner in different environments (Fig. 3; Fig. S6). For example, an increase in the AD5 viral titer enhanced the fluorescence signal of the mixture with 5 μM PBET (Fig. 3a and b; Fig. S6i); the peak intensity at 510 nm showed a good linear correlation (R^2^ = 0.9904) with the viral titer in the range of 10^5^–10^7^ TCID_50_ mL^−1^ (Fig. 3c). These findings suggest that this labeling strategy can be used for the quantitative analysis of viruses. Similar fluorescent labeling was achieved for non-enveloped EV-D68 viruses (Fig. 3e through g; Fig. S6a[ii]) and enveloped SEV viruses (Fig. 3i through k; Fig. S6a[iii]). No significant changes in targeting or responsiveness were observed under different physiological environments. What’s more, regardless of this permutation of external environmental changes, the fluorescence signals of the sensor were not affected, which confirms that the sensor is environmentally insensitive in achieving virus targeting, switching on the fluorescence switch, and virus-compatible attachment processes.

To further test the applicability of PBET in virus imaging, we labeled a variety of viruses after purification. It was found that virus particles at low concentrations rapidly attracted free PBET in the PBET solution, eventually showing distinct fluorescence and revealing a perfect overlap of PBET and virus particles. By a simple incubation protocol, a PBET sensor was possible without a significant background signal (Fig. 3d: AD5; Fig. 3h: EV-D68; Fig. 3l: SEV). We also found that the virus switched on the fluorescence with high efficiency, with the response reaching a steady state instantaneously (<1 min) after mixing. This experiment confirmed that all three viruses can be labeled stably to the maximum extent possible under our imaging conditions.

### Nondestructive infestation of PBET-tagged viruses

**Fig 4:**
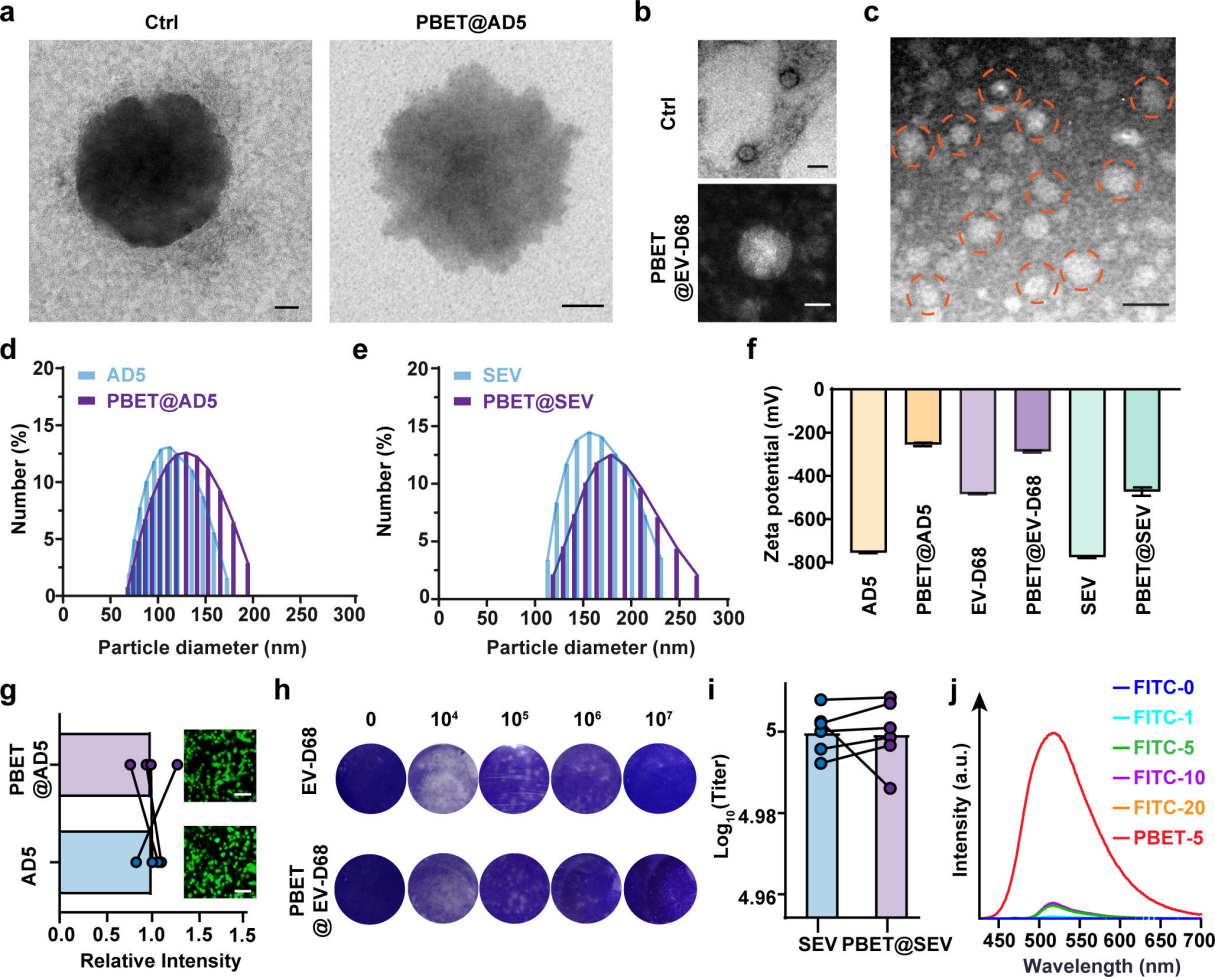
Nondestructive labeling process of the PBET sensors. (**a**) TEM characterization of AD5 before and after labeling with PBET. Scale bar: 50 nm. (**b**) TEM characterization of EV-D68 before and after labeling with PBET. Scale bar: 100 nm. (**c**) TEM characterization of the SEV after PBET labeling. Scale bar: 100 nm. (**d**) DLS measurement of AD5 before and after labeling with PBET. (**e**) Particle size distribution of the SEV before and after labeling. (**f**) The surface zeta potential of virus particles before and after labeling with PBET was measured via DLS. [PBET] = 5 µM. (**g**) Quantification of transfection by loading the EGFP plasmid AD5 virus with PBET before and after mixing with PBET in the same cell mixture; fluorescence images show green fluorescence expression after transfection; scale bar: 50 µm. (**h**) Empty spot experiment of PBET mixed with EV-D68. (**i**) Titer test of a PBET mixed with SEV. (**j**) Fluorescence curves of FITC-Linker-labeled viruses or PBET fluorescently labeled complexes at different concentration gradients. [PBET] = 5 µM.

Previously, we reported that current viral tracer labels can affect the virus particles themselves, for example, affecting their morphology. Thus, in our experiments, we characterized the morphology of virus particles before and after PBET sensor labeling. The results revealed that the labeling of the sensors did not significantly change the size of the virus particles. Importantly, the characteristic morphology and size of AD5 and EV-D68 did not change significantly (Fig. 4a for AD5; Fig. 4b for EV-D68). Meanwhile, the results of transmission electron microscopy revealed the classical morphology of the enveloped virus SEV (Fig. 4c).

Dynamic light scattering (DLS) was used to characterize the virus particles in solution before and after PBET labeling. The particle size distributions of the three labeled virus samples were similar to those of the corresponding unlabeled virus samples, with only a slight increase in the average peak size (Fig. 4d and e; Fig. S7a). At the same time, we confirmed that under standard labeling conditions, the surface zeta potential (ξ) of the virus particles fluctuated slightly after treatment with the dye but remained negatively charged (Fig. 4f): from −755 mV to −255 mV for AD5, from −484 mV to −287 mV for EV-D68, and from −776 mV to −472 mV for SEV. These slight fluctuations in surface charge could imply that the presence of viruses and sensor connections is associated with electrostatic adsorption.

To address potential issues with existing fluorescent labeling methods, we conducted a more in-depth investigation into the impact of PBET visualization on viral particle infectivity. First, we measured the titer and transfection capacity to compare the infectivity of labeled and wild-type virus batches. The transfection efficiency of AD5 particles was validated using commonly used transfection vectors to determine whether viral transfection capacity was affected before and after labeling. After loading the GFP plasmid and incubating with HEK 293A cells, green fluorescence in HEK 293A cells was detected using confocal microscopy. The results showed no significant reduction in transfection efficiency for PBET-labeled AD5 particles (Fig. 4g; Fig. S7b). Moreover, the wild-type EV-D68 virus with a titer of 10^7^ TCID_50_ mL^−1^ was selected, labeled with PBET, and then subjected to a viral titer assay (Fig. 4h; Fig. S7c). Both viruses were able to detect virus-induced vacuoles in 10^−7^-well plates. This process, moreover, can also be demonstrated by nondestructive labeling during the injection of enveloped viruses such as SEV (Fig. 4i; Fig. S7d). Moreover, compared to the FITC or the QD labeling strategy, PBET demonstrated superior viral labeling minimum concentrations and maximum fluorescence release intensities (Fig. 4j; Fig. S7d).

### Positively charged chemotactic binding mechanism between PBET and viral proteins

**Fig 5:**
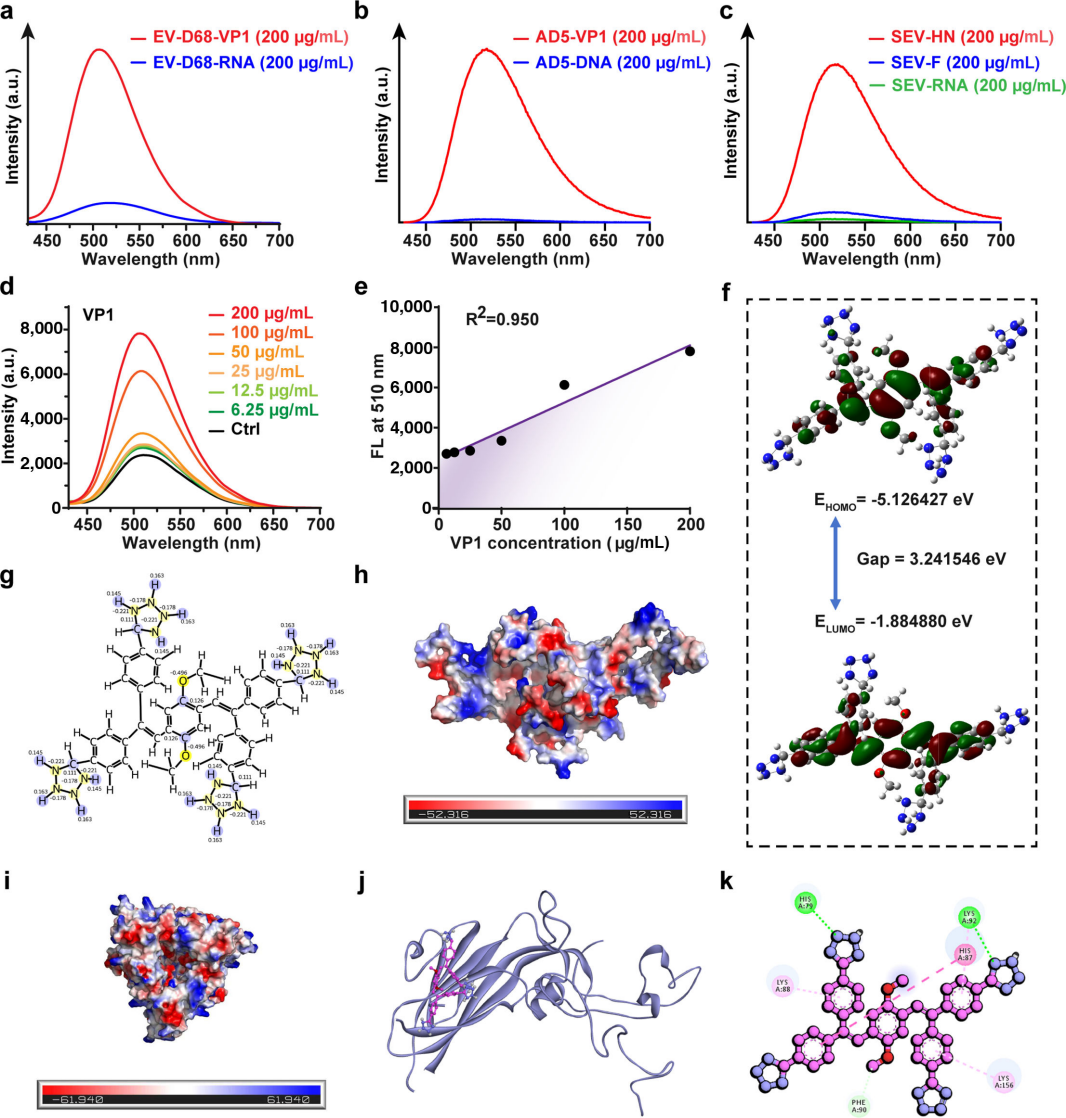
Positive charge chemotactic binding mechanism between PBET and viral proteins. (**a**) Quantitative fluorescence curve analysis of labeled products formed between PBET and different EV-D68 components. (**b**) Quantitative fluorescence curve analysis of labeled products formed between PBET and different AD5 components. (**c**) Quantitative fluorescence curve analysis of labeled products formed between PBET and different SEV components. (**d**) Quantitative fluorescence curve analysis of labeled products formed by PBET with EV-D68 VP1 at gradient concentrations. (**e**) Fitting curve between fluorescence emission peak at 510 nm and VP1 concentration in (**d**). (**f**) Calculated HOMO/LUMO values for the PBET sensor. (**g**) Calculated charge distribution of the PBET sensor molecule in aqueous solution. (**h**) Surface potential distribution of the VP1 from the AD5 virus. (**i**) Surface potential distribution of the F protein analog from the SEV virus. (**j**) Molecular docking simulation between the PBET sensor and EV-D68 VP1. (**k**) Molecular interactions formed between PBET and EV-D68 VP1 within the docking pockets. [PBET] = 5 µM.

Based on the unique charge properties of viral protein surfaces, we designed and constructed the PBET sensor. To validate the binding mechanism between PBET and viral proteins, we first performed a series of spectroscopic analyses by forming fluorescently labeled complexes with PBET sensors and various viral components. Experimental results clearly demonstrate that during PBET binding to EV-D68 viral components, VP1 protein exhibits a significantly high affinity for the PBET sensor (Fig. 5a). Similarly, in PBET binding experiments with purified AD5 components, the capsid protein also showed significantly higher affinity than the DNA component of the AD5 viral vector (Fig. 5b). Furthermore, multiple types of binding proteins exist on the membrane surface of enveloped viruses. Taking SEV as an example, both the fusion protein (F protein) and neuraminidase protein (HN protein) present on the viral surface were tested as PBET binding substrates via fluorescence intensity measurement. The results revealed that the F protein in SEV exhibited significantly higher fluorescence release than other proteins and nucleic acid components (Fig. 5c). Based on these findings, we propose that PBET sensor labeling may correlate with the surface protein interface properties of viral particles. Notably, in spectral experiments where purified components bind independently, proteins still exhibit significantly stronger binding affinity compared to nucleic acids within the viral particle. Consequently, we conducted in-depth research on this PBET labeling mechanism for viral proteins as the core function of the PBET sensor. First, PBET binding to viral proteins exhibits concentration dependence. As VP1 concentration increases, fluorescence intensity significantly enhances, showing good linear correlation (R^2^ = 0.950, Fig. 5d and e). This concentration-dependent fluorescence increase typically arises from the highly reactive nature of the substrate-binding interface. Therefore, integrating existing analyses and studies of viral protein particles, we focused the PBET binding site on positively charged pockets on the viral protein surface. We first calculated the electronic charge distribution of PBET, revealing that the introduction of the tetrazole group confers a negative charge to the PBET molecule (Fig. 5f). The tetrazole-modified CEOCH, similar to the fluorescent core, possesses an electronic transition space and can release fluorescence during aggregation (Fig. 5g). Subsequently, we simulated the surface potentials of AD5, SEV, and EV-D68 proteins, identifying positively charged aggregation pockets on their surfaces (Fig. 5h through j). We selected EV-D68, exhibiting the most complex charge state and pocket structure, as the target protein for molecular docking. The results revealed that the PBET sensor binds to the core positively charged pocket of the protein, forming stable intermolecular interactions (Fig. 5k; Fig. S8a through h).

In summary, the electrostatic adsorption between the PBET sensor and viral particles underpins the stable labeling of viral particles by various fluorescently labeled complexes such as PBET@Virus. The designed PBET molecule achieves stable tracing by binding to positively charged aggregation pockets on the viral surface while also facilitating the aggregation-induced luminescence of PBET molecules.

### Unique molecular-induced targeted buffering mechanism enhances binding capacity

**Fig 6:**
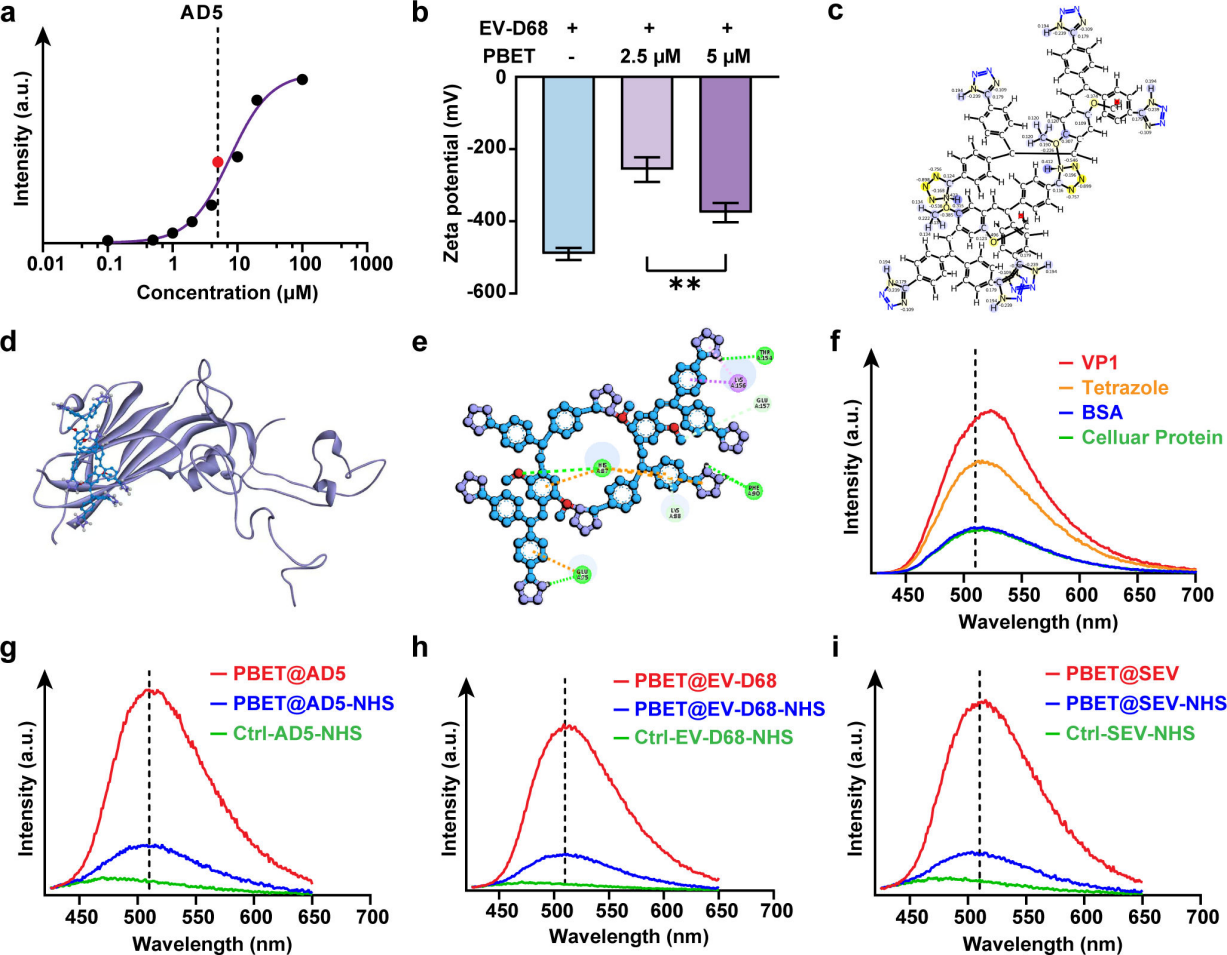
Molecular-induced targeted buffering mechanism enhances binding capacity. (**a**) Regression curve of the 510 nm emission peak for PBET forming fluorescently labeled complexes with AD5 virus particles under concentration gradients. [Virus] = 10^7^ TCID50 mL^−1^. (**b**) Potential changes in different concentrations of PBET mixed with EV-D68. **P < 0.01. (**c**) Intramolecular atomic charge enhancement by dimeric PBET. (**d**) Molecular docking simulation between dimeric PBET and EV-D68 VP1. (**e**) Intermolecular interactions formed by dimeric PBET within the VP1 docking pockets. (**f**) Fluorescence spectra of PBET-labeled viral protein complexes incubated with different competitive substances. VP1: Fluorescence spectrum of VP1 fluorescently labeled viral protein complex. Tetrazole: Fluorescence spectrum of VP1 fluorescently labeled complex after incubation with etrazole-mPEG. BSA: Fluorescence spectrum of BSA solution after incubation with magnetic bead-immobilized PBET@VP1. Cellular Protein: Fluorescence spectrum of cellular protein solution after incubation of RD cell total protein extract solution with magnetic bead-immobilized PBET@VP1. (**g**) Fluorescence spectra of AD5 labeled with PBET under positively charged amino acid shielding. (**h**) Fluorescence spectra of EV-D68 labeled with PBET under positively charged amino acid shielding. (**i**) Fluorescence spectra of SEV labeled with PBET under positively charged amino acid shielding. [PBET] = 5 µM.

The positively charged aggregation pockets on the viral surface provide binding sites for the PBET sensor. However, the aggregation of small molecules not only alters the surface properties of the protein but also leads to the separation of polymers from the protein, thereby generating false signal sources. To further evaluate PBET molecule stability and reliability, fluorescence peak curves were obtained at different PBET concentrations after viral labeling via PBET concentration gradient fitting. The results revealed that during PBET labeling, the fluorescence peak exhibited a three-segment binding range with increasing concentration (Fig. 6a; Fig. S9). Taking AD5 as an example, experiments revealed that PBET struggles to adsorb and aggregate with viral proteins at low concentrations. However, once concentrations exceed a threshold (approximately 2–3 μM), the concentration-dependent fluorescence intensity of PBET-labeled viruses increases exponentially, peaking at 5 μM. Further increasing PBET concentration does enhance the number of fluorescent molecules attached to viral proteins, but the growth rate gradually diminishes. Moreover, when PBET concentration exceeds 100 μM, the molecules undergo self-aggregation-induced luminescence due to the dramatic increase in local molecular density. This super-aggregation-induced fluorescence interferes with the characterization and analysis of the fluorescently labeled complexes. By characterizing different regions, we selected 0, 2.5, and 5 μM as representative values for viral particle potential analysis after labeling (Fig. 6b). The results indicate that when PBET in a low-aggregation state adsorbs onto the positively charged aggregation pockets on the viral surface, it elevates the surface charge of viral proteins. Interestingly, however, the surface charge of viral protein particles bound to 5 μM PBET decreased. We propose this may result from intramolecular interactions within the CEOCH fluorescent core and intermolecular interactions between PBET molecules, ultimately forming a dimeric compound (Dual-PBET). Moreover, this polymeric form enhances molecular charge transfer effects, establishing a protective mechanism akin to a buffer solution within the viral protein binding region. We term this mechanism molecular-induced targeted buffering (MITB). Previous studies indicate that intermolecular aggregation forms polymeric structures and enhances functionality (73). Therefore, through molecular modeling and molecular simulation experiments, we verified that PBET can form dimers in aqueous solution, significantly enhancing the negative charge of the N atom in the tetrazole group (Fig. 6c). The formation of this Duel-PBET conformation reduces the energy required for PBET molecules to bind proteins (while maintaining the same number of hydrogen bonds), enabling the formation of more hydrogen bonds at the same energy cost. This enhances the fluorescence intensity of PBET molecules upon activation (Fig. 6d and e). Based on this experimental prediction, analysis of other molecules in the fluorescent library revealed that multiple tetrazole groups can indeed restrict the free movement of fluorescently labeled molecules, thereby inhibiting excited-state energy dissipation pathways and triggering fluorescence-on sensing. As described in the initial screening, among over ten thiazole-modified fluorescent probes, only PBET exhibited a robust fluorescence-on response toward viruses. We validated and provided evidence that PBET enters and binds to the positively charged aggregation pocket structure of viral proteins in a specific manner. The PBET sensor directly binds to the positively charged aggregation pocket structure via electrostatic adsorption, labeling the virus. Simultaneously, at appropriate concentrations, PBET dimers (or polymers) form complexes that enhance fluorescence. This process requires neither prior chemical modification of viral cells nor cumbersome labeling strategies like antibodies, offering an attractive approach for future studies tracking viral infection.

Furthermore, PBET employs electrostatic adsorption for protein labeling during the tagging process. Once these viral proteins enter the cellular environment, they inevitably interact with other types of proteins present and carry a potential risk of dye detachment. To validate the stability and target affinity of the PBET sensor during this process, we performed multi-protein labeling. Incubate PBET-labeled viral proteins with serum proteins or cellular proteins and observe the fluorescence intensity of different protein-labeled complexes. The results revealed that during binding to different proteins, PBET exhibited extremely high preference for viral proteins (Fig. 6f). Compared to the rapid labeling and high fluorescence intensity observed with viral proteins, the PBET sensor exhibited lower sensitivity toward animal-derived proteins. We hypothesize this difference stems from the distinct electrostatic adsorption capabilities: viral proteins possess positively charged aggregation regions evolved for target cell infection, whereas cellular proteins exhibit widespread negative surface potentials. Due to surface potential, cellular proteins likely interfere with PBET sensor intermolecular interactions via electrostatic repulsion. This increases PBET dispersion in aqueous solutions and induces molecular distortion. The altered aggregation state of PBET in aqueous media induced by animal proteins ultimately prevents PBET sensor localization to animal cell proteins, conferring reliability for visualizing viral infection. Furthermore, through competitive experiments, we pre-filled or shielded the positively charged regions of viral proteins and verified whether the treated viral proteins could bind to the PBET sensor to form a fluorescently labeled complex (Fig. 6f through i). The results showed that the fluorescent labeling capacity of the PBET sensor was competitively inhibited by free tetrazolium, reducing its viral labeling ability. Furthermore, shielding the side chains of positively charged amino acid residues on the viral protein surface significantly diminished the PBET sensor’s fluorescent labeling capacity. In experiments where NHS-mediated covalent bonding was used to link the amino groups on the side chains of arginine and lysine residues in viral proteins, multiple surface proteins from AD5, EV-D68, and SEV demonstrated shielding inhibition.

Collectively, we demonstrate the unique binding mechanism of the PBET sensor during its interaction with viral proteins. The fluorescence labeling and complex formation processes observed in our experimental data enable stable labeling of target proteins through strong electrostatic attraction. Simultaneously, the molecule’s intrinsic electron and proton transfer capabilities provide a buffer-like MITB protection mechanism at the binding site. This enables targeted fluorescent aggregation at the positively charged pocket of viral proteins without compromising the virus’s ability to bind to cellular receptors. Specifically, we have also worked to avoid abnormal staining or non-viral protein staining errors during PBET-based virus tracing *in vivo* or *in vitro*. Through data simulation and spectral characterization of the structural basis, evolutionary process, and post-binding fluorescence expression with cellular proteins, we demonstrate that the PBET sensor is an efficient, stable, and reliable tool for virus visualization.

### Visualization tools for real-time tracking of viral infection in cells

**Fig 7:**
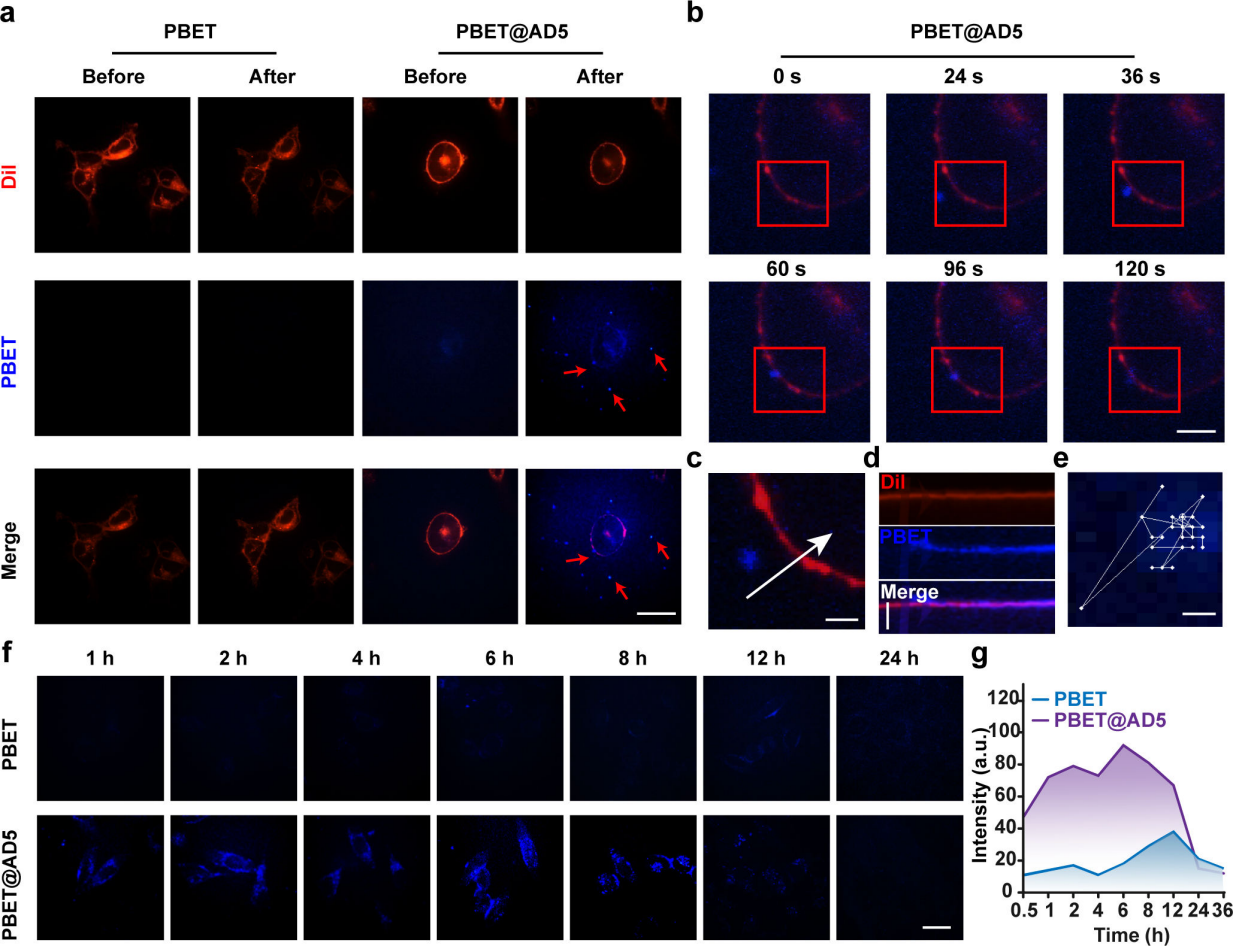
Fluorescence tracking analysis of PBET-labeled AD5 virus particles in living 293A cells. (**a**) Confocal fluorescence image of cells after incubation with labeled AD5 virus. Red: Dil-labeled cell membrane; blue: PBET-labeled AD5 virus particles. Scale bar: 25 µm. (**b**) Video snapshot of PBET-labeled virus entering the cell taken by confocal fluorescence imaging; scale bar: 5 µm. (**c**) Superimposed image of changes over time at the viral binding site, with arrows indicating the direction of viral binding. Scale bar: 1 µm. (**d**) Kymograph of the PBET-labeled virus binding site in part (**c**). Scale bar: 12.5 nm. (**e**) Viral diffusion trajectory based on the continuous image in part (**b**). Scale bar: 1 µm. (**f**) Long-term fluorescence tracking of PBET-labeled AD5 viruses in live 293A cells; controls were generated with virus-free PBET dye solutions. Scale bar: 25 µm. (**g**) Fluorescence retention curve of the PBET-AD5 virus over time in (**f**).

Tracking viruses in the extracellular space provides a direct visualization of viral particle infection processes and strategies. To achieve real-time imaging of various viruses in the extracellular space, the cells were mixed with fluorescently labeled viral complexes. Specifically, the cells were first incubated with labeled viruses for 30 min and observed under a fluorescence microscope after washing with fresh DMEM. Numerous emitting viral particles (highlighted by arrows) were identifiable, localized to membranes (red, Dil-stained) adjacent to 293A cells (Fig. 7a). In other experiments, the fluorescence of EV-D68 and SEV virus particles was comparable (Fig. S10a and S11a). These results indicate that the labeling strategy does not interfere with virus binding to host cells. Conversely, in control experiments, particles emitting blue-green light could not be detected when PBET was added directly to the cell culture mixture.

Real-time tracking analysis was performed to visualize the attachment, entry, and intracellular translocation of PBET-labeled viruses in living cells (Fig. 7b for AD5; Fig. S10b for EV-D68; Fig. S11b for SEV). The infection process of individual viruses was monitored at 12 S intervals. We captured a representative snapshot accurately showing a virus particle located on the cell membrane (Fig. 7c). Kymograph plots of PBET-labeled viral binding sites visualize the asymptotic progression of viral particles in the vicinity of membrane target cross sections, further confirming the co-localization of viral fluorescence (Fig. 7d). The trajectory of this single virus throughout the process was extracted from the video, which shows the movement of the virus in the peripheral space/cell membrane prior to internalization (Fig. 7e).

By tracking the fluorescence images of the PBET-labeled virus for 36 h after incubation, we monitored the course of viral movement throughout the early postinfection period. Fluorescently labeled AD5 viruses were observed on the immediate cell membrane after incubation with 293A cells at 37°C and accumulated over time, reaching a maximum at 2 h, as indicated by the corresponding fluorescence intensity (Fig. 7f and g). Subsequently, the localization of viral particles on the membrane gradually diminished and shifted into the cytoplasm. Fluorescence visualization and fluorescence intensity curves indicated that the fluorescence of viruses on the cell membrane progressively weakened and persisted for 8–12 h, with a fluorescence-labeled single-cell half-life of approximately 10 h. During the fluorescent labeling visualization cycle, three distinct phases can be identified: membrane-associated fluorescence beginning at 1 h, cytoplasmic fluorescence following viral particle internalization starting at 2 h, and the attenuation process during which loosely bound viral particles are cleared between 8 and 12 h (Fig. 7g). At this stage, viruses can remove the envelope and/or capsid, release their DNA or RNA, and enter the hidden infection phase. The loss of virus-associated fluorescence is primarily attributed to the degradation of PBET-labeled viruses, as well as the dissolution and clearance of PBET within the cytoplasm. As confirmed by prior competitive experiments, the charge properties of PBET make it difficult for fluorescence to be re-released through binding to cytoplasmic proteins. Consequently, the PBET-tracing process perfectly elucidates the movement trajectories of cells during the early stages of viral infection. Furthermore, we performed visual verification of cellular infection for other enveloped and non-enveloped viruses. Similar to AD5, as the capsid and/or envelope are stripped away, and the PBET dye detaches from viral proteins (especially when these proteins are digested and/or degraded by the cell) and becomes solubilized, resulting in diminished fluorescence. We observed a similar phenomenon when PBET-labeled EV-D68 and SEV viruses were used to infect different types of cells (Fig. S10f, S11e and f). As described above, these viruses bind to receptors on the cell membrane within 1 h of incubation with the cells and then endocytose and form endosomes in the cytoplasm within another hour. After 8-12 h of infection, the viral envelope is largely absent, and the virus begins to enter the latent phase of replication. The infection process of these labeled viruses is broadly similar to that reported in the literature.

### Localizing the primary viral infection site in experimental animals

**Fig 8:**
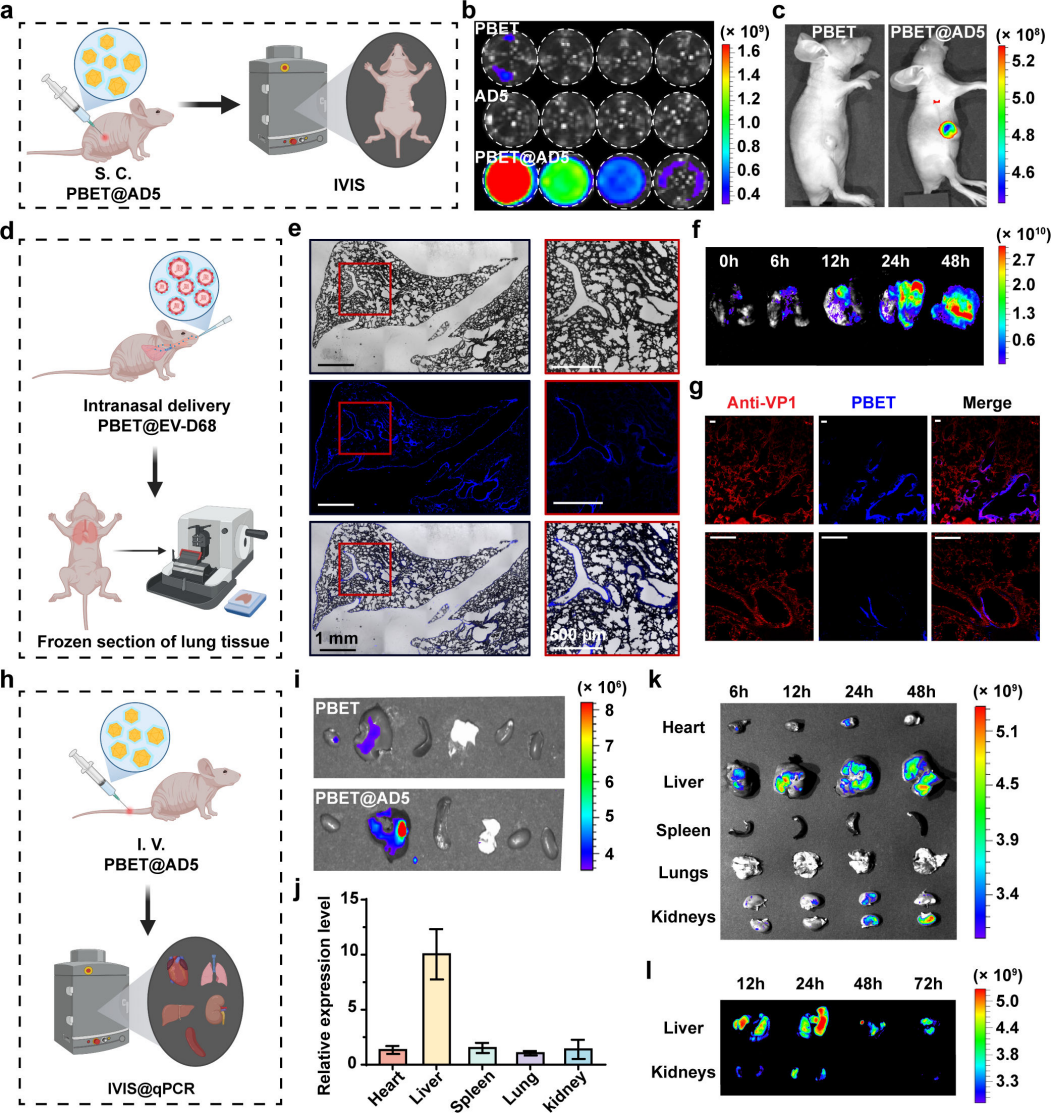
Fluorescent bioimaging of different animal virus infection models. (**a**) Schematic of the PBET transdermal visualization protocol and experimental characterization in animals. (**b**) Fluorescence images of sample wells captured using an *in vivo* imaging system, with fluorescence excitation achieved via a 420 nm narrow-band filter. (**c**) Fluorescent bioimaging of mice following subcutaneous injection of PBET@AD5 fluorescently labeled complexes. (**d**) Establishment and visualization protocol for the nude mouse respiratory infection model. (**e**) Fluorescent scans of *ex vivo* isolated lung tissue from the pulmonary infection model, with PBET-labeled viral particles displayed in blue. (**f**) Fluorescence imaging of *ex vivo* lung tissue at different infection time points. (**g**) Immunofluorescence confocal images of *ex vivo* lung tissue at 24 h. Red: Viral particles labeled with rhodamine B-tagged rat anti-EV-D68 antibody. Blue: PBET-labeled EV-D68 viral particles. Scale bar = 150 µm. (**h**) Nude mouse blood infection model establishment and visualization protocol. (**i**) Fluorescence images of organs harvested 12 h after tail vein injection of different PBET@virus samples. (**j**) Relative viral load intensity in organs harvested 12 h after tail vein injection of PBET-labeled AD5, measured by RT-qPCR. (**k**) Fluorescent visualization of *ex vivo* organs after sequential tail vein injections at different time points. Organs were obtained from animals euthanized uniformly at 48, 24, 12, and 6 h after pre-injection. (**l**) *Ex vivo* fluorescence imaging of liver and kidney 12-72 hours after tail vein injection of pure PBET sensor solution. All schematic diagrams created with BioRender.com, with permission.

For potential pathogens or large-scale unknown viral infections, we envision utilizing PBET-labeled purified viruses in animal models to advance drug development and mechanism studies. We evaluated these PBET-labeled viruses *in vivo* bioimaging studies using mouse models. Despite challenges in obtaining clear images *in vivo* with this UV-fluorescent technology, we validated a potential *in vivo* fluorescence visualization approach for PBET through multiple *in vivo* models (Fig. 8a). Here, we performed fluorescence signal imaging of different PBETs, viruses, and fluorescently labeled complexes using an *in vivo* imaging system (IVIS). The results demonstrated significantly enhanced signal intensity for multiple viruses (Fig. 8b; Fig. S12a). Subsequently, we subcutaneously injected viruses into three groups of mice. Long-term exposure revealed strong fluorescent signals at the injection sites in BALB/C nude mice infected with PBET-labeled viruses, whereas no such signals were observed in the subcutaneous injection sites of mice treated with pure PBET solution (Fig. 8c; Fig. S12b).

Furthermore, we successfully established a BALB/C nude mouse model for nasal droplet infection with the virus, enabling early-stage monitoring of physiological status. Following successful modeling, we obtained post-infection lung tissue (Fig. 8d). Significant fluorescence signals were detected in lung tissue sections from infected mice, co-localizing with bronchioles, alveoli, and the pulmonary surface (Fig. 8e; Fig. S13). Concurrently, fluorescence visualization imaging of *ex vivo* lung tissue obtained at different time points revealed that PBET-labeled virus persisted along the trachea and esophagus from 0 to 12 h post-infection. As time progressed, high viral concentrations migrated into the bronchi, resulting in extensive fluorescence distribution in lung tissue, particularly in the right upper lobe, at 12–24 h post-infection. Longer follow-up revealed gradual fluorescence reduction in the initial infection sites, accompanied by enhanced fluorescence release in the lobes (Fig. 8f). To address challenges in *in vivo* fluorescent visualization of viral presence, we conducted a series of characterizations and analyses to provide robust evidence supporting PBET as a reliable tracer molecule in pulmonary infection models. Consequently, we performed further fluorescent visualization and colocalization experiments with viral protein antibodies on lung tissue sections. The results directly demonstrate that the fluorescently labeled complexes bound by the PBET sensor primarily localize on the surface of bronchioles. Visualization images clearly show strong colocalization between the blue-green fluorescence emitted by PBET and the red fluorescence emitted by rhodamine B-labeled anti-EV-D68 (Fig. 8g). These data demonstrate that PBET serves as a visualizable tool for lung virus binding in animal models, revealing viral binding sites, binding regions, and druggable targets.

Furthermore, we designed a BALB/C nude mouse model for the bloodstream infection using PBET sensors. Different groups of PBET were injected into BALB/C nude mice via the tail vein, and the mice were euthanized 12 h post-injection (Fig. 8h). Major visceral organs were visualized using an *in vivo* imaging system, and organ samples underwent quantitative real-time polymerase chain reaction (RT-qPCR) to quantify viral accumulation (Fig. 8i through k). The results revealed that the AD5 virus primarily targeted hepatocytes in blood-borne infection, with the PBET@AD5 group exhibiting more precise hepatic fluorescence distribution compared to the pure PBET group. Combined with tissue RT-qPCR results, we found good correlation between viral accumulation patterns and bioimaging findings, consistent with the binding mechanism at viral infection targets. Furthermore, we analyzed the potential metabolic and clearance patterns of PBET *in vivo*. Intravenously injected PBET molecules gradually initiated hepatic clearance after 24 h and were eliminated via hepatic and renal metabolism by 48 h (Fig. 8l). Although the imaging cycle of PBET molecules at the cellular level is 1–12 h, the entry of high-concentration viral particles and their recognition/binding cycle to target organs *in vivo* imaging are significantly longer than those *in vitro* simulations. Similar to the time-dependent fluorescence decay of PBET-fluorescently labeled complexes in serum-containing media during experiments, the tracer window of PBET in *in vivo* visualization models enables precise visualization over 24–48 h (Fig. S14). The *in vivo* visualization timeframe demonstrated by PBET not only supports early-stage viral fluorescence localization but also significantly exceeds the half-life under *in vivo* clearance pathways and serum conditions.

In summary, we propose a sensing platform capable of imaging live viruses in the vicinity of living cells and *in vivo* environments. This sensor leverages electrostatic adsorption via positively charged aggregation pockets and molecularly induced buffering effects to achieve nondestructive binding and electrostatic adsorption to viral surface proteins. Using existing viral tracing methods as controls, we validated the superior suitability of our designed sensor for viral labeling. Using molecular docking to elucidate binding regions and aggregation-enhanced binding mechanisms, we demonstrate that PBET enables efficient, stable, and persistent visualization of viruses. These findings also inform the design of more stable near-infrared fluorescent dye cores and safer, more robust targeted sensors. Since existing aggregation-emitting dyes predominantly exhibit green fluorescence, *in vivo* imaging for biological tracing remains challenging. Future development of PBET-based viral tagging sensors aims to achieve *in vivo* tracing of viral infection processes in nude mice without ex vivo sampling by selecting optimized far-infrared excitation cores.

## DISCUSSION

In this study, we report a fluorescently labeled sensor (PBET sensor) based on protein-based enhanced targeting (PBET) and molecularly induced targeting buffer (MITB) technology for viral tracing analysis. This technique is simple, efficient, and reproducible, with minimal interference to viral infectivity without requiring prior modification of viral particles. Such sensors can be widely applied to both enveloped and non-enveloped viral strains. Fluorescent labeling is achieved by simply mixing the PBET dye solution with the viral sample. Experimental and computational studies indicate that this unique labeling occurs through electrostatic adsorption of PBET molecules onto the outermost proteins of the viral capsid or envelope. Due to positively charged pockets formed during viral particle evolution, PBET molecules can enter these protein residue pockets and aggregate to emit light.

Further particle characterization revealed that the morphology and physical aggregation state of PBET-labeled viruses were largely preserved. As sensor concentration increased, the electrostatic binding-induced changes in viral surface charge were mildly restored, indicating that this process achieved a buffering effect through sensor-mediated aggregation. We subsequently employed PBET-labeled viruses for proof-of-concept studies, enabling real-time observation of viral infection in susceptible live cells over a 24-h timeframe. These findings demonstrate the versatile application potential of PBET-labeled viruses. Furthermore, PBET-labeled viruses are compatible with *in vivo* animal models, facilitating viral studies in living biological systems: they showcase infection processes in different models while revealing the potential for *in situ* analysis of viral accumulation within viral models.

The findings of this study align with previous technical research, but its innovative targeting and buffering design represent a revolutionary breakthrough in the field of nondestructive, high-efficiency viral labeling. However, this research still has limitations. In experiments, we observed that the fluorescence emitted by PBET molecules during binding is blue-green light with an excitation wavelength of 405 nm. This makes it challenging to achieve transdermal visualization or visualize viral trajectories within multilayered cells. Future sensor designs will focus on developing more sensitive red-shifted excitation wavelength modules, utilizing core-shell luminescent dyes with excitation wavelengths exceeding 550 nm to enable *in vivo* viral tracking and target exploration (74–76). This paper proposes that the constructed PBET represents a visualization solution capable of rapidly verifying viral trajectories and identifying infection zones during future unknown viral outbreaks. However, during PBET design and experimentation, obtaining purified viral particles from highly transmissible and virulent viruses proved challenging. Consequently, the study extensively utilized well-studied viral types, limiting the ability to identify novel viral targets during the viral labeling process. Future experiments will focus on partially characterized viruses, leveraging the charge properties within PBET sensors to track and localize viral particles in extracellular spaces. Consequently, the study extensively utilized well-studied viral types, limiting the ability to identify novel viral targets during the viral labeling process. Future experiments will focus on partially studied viruses, leveraging the charge properties within PBET sensors to track and localize viral particles in extracellular spaces. The next phase of PBET development will explore whether labeled viral particles can serve as selection targets for separating target proteins using fluorescent particles and implementing screening and identification of viral receptor proteins. This potential separation strategy, combined with future co-localization of antibody dyes with viral labels, will significantly advance the identification of membrane receptor molecules for unknown viruses. Additionally, the paper highlights PBET’s advantages over traditional fluorescent labels, though challenges remain in molecular synthesis and stability. Regrettably, commercially available viral labeling tools based on quantum dots or green fluorescent dyes struggle to replicate the highly efficient methods and long-term tracking capabilities demonstrated in this study. Consequently, the paper only compares the covalent quantum dot-virus labeling approach with the FITC protein labeling method to illustrate PBET’s superior stability and reliability. Mechanistically, PBET’s luminescence relies on hindered electron transfer triggered by molecular aggregation. Consequently, its fluorescence visualization duration depends on the maximum aggregation time of fluorescent molecules on the protein surface. Once polymeric complexes achieve long-term stable attachment to the protein surface, the tracking time of the fluorescent dye can be significantly extended. Compared to quantum dot and fluorescent protein labeling, this offers fundamental differences in tracking duration and fluorescence stability. Furthermore, PBET exhibits an extremely low molecular weight with negligible volume, unlike the inherent bulkiness of quantum dots or the steric hindrance of green fluorescent proteins. No visible self-assembled fluorescent complexes were observed during the aggregation-emission process. This eliminates concerns about protein masking or steric hindrance changes during PBET visualization of virus labeling. Experimental data demonstrate that PBET-labeled viral particles exhibit no significant differences from native viruses in morphology, size, surface charge, or infectivity. Thus, both theoretical and experimental results confirm PBET as an efficient, stable, and interference-free virus visualization strategy. In summary, the design and synthesis of PBET molecules offer a novel approach to viral labeling based on electrostatic adsorption and binding to positively charged pockets. Although room for improvement remains, the current PBET molecules demonstrate applicable potential in various viral tracking processes.

In conclusion, this strategy not only provides very valuable traceability of viruses but also maximizes the fluorescence properties of the live sensor and the infectivity of the virus, which is essential for advanced imaging and analysis of viral particles at different cellular, organ, and animal levels. Importantly, and interestingly, this strategy is a versatile and specific molecular tool for labeling enveloped and unenveloped viruses, especially wild-type strains. Therefore, this tool may have immediate application in the study of antiviral-associated infectious diseases. Future application of this technology is expected to allow visual validation of virus-targeting studies at the cellular level and in animal models. With the development of detection technologies for unknown pathogens, this sensor will provide revolutionary solutions for virology and even pathogenic microbiology.

## Data Availability

All data needed to evaluate the conclusions in the paper are included in the paper and its supplemental material. For any other inquiries, please contact the corresponding author.

## SUPPLEMENTAL MATERIAL

Supplemental material jvi.00044-26-s0001.pdf Supplemental text and Fig. S1 to S14.

ASM does not own the copyrights to Supplemental Material that may be linked to, or accessed through, an article. The authors have granted ASM a non-exclusive, world-wide license to publish the Supplemental Material files. Please contact the corresponding author directly for reuse.
