# Supplementary material for: Nondestructive tracking of viral infections by viral protein-initiated fluorescent sensors
Source: J Virol. 2026 Mar 17;100(4):e00044-26. doi: 10.1128/jvi.00044-26 (PMC13098219; doi:10.1128/jvi.00044-26)
Supplement: Supplemental material — Supplemental text and Fig. S1 to S14. [file jvi.00044-26-s0001.pdf]

Supplementary information

Nondestructive tracking of viral infections by viral protein-initiated fluorescent sensors

Zuoxi Zhang[1,2,#], Xianhuang Li[1,#], Kexin Yin[1,2,#], Chunguang Yin[3], BowenLi[1], Wenchang Peng[1], Jinsong Han[4], Boyu Luo[5], Yue Teng[5,*], Sheng Xie[3,6,*], Tao Wang[1,*], Bin Zheng[1,2,*]

[1] Faculty of Medicine, Tianjin University, Tianjin, China.

[2] The province and ministry co-sponsored collaborative innovation center for medical epigenetics, School of Biomedical Engineering and Technology, Tianjin Medical University, Tianjin, China.

[3] State Key Laboratory of Chemo and Biosensing, College of Chemistry, Hunan University, Changsha, China.

[4] State Key Laboratory of Natural Medicines, National R&D Center for Chinese Herbal Medicine Processing, Department of Food Quality and Safety, College of Engineering, China Pharmaceutical University, Nanjing, China.

[5] State Key Laboratory of Pathogen and Biosecurity Beijing Institute of Microbiology and Epidemiology, Academy of Military Medical Sciences, Beijing, China.

[6] Shenzhen Research Institute of Hunan University, Hunan University, Shenzhen, China.

[#] These authors contributed equally to this work.

[*] Corresponding authors.

E-mail addresses: yueteng@me.com (Y. Teng), shengxie@hnu.edu.cn (S. Xie), wangtaobio@tju.edu.cn (T. Wang), binzheng@tju.edu.cn (B. Zheng).

Table of contents:

1. General Information

2. Experimental Procedures

2.1 Animal Ethical Statement

2.2 Sensor Preparation

2.3 Characterization

2.4 Preparation of Virus for Labeling

2.5 Virus Labeling Protocol

2.6 Modeling of Molecular Docking

2.7 Real-time qPCR

2.8 $TCID_{50}$ assay

2.9 Fluorescence in Vivo Bioimaging

2.10 Image Analysiss

3. Supplementary Tables 1-2

4. Supplementary Figures 1-14

5. References

1. General Information

All chemicals were commercial reagent grade and used without further purification, unless otherwise stated. The adenovirus serotype 5 (AD5) strain was a kind gift from Prof. Zhuozhuang Lu's group at the Chinese Center for Disease Control and Prevention. The Sendai virus (SEV) BB1 strain was a kind gift from Dr. Lishu Zheng's group at the Chinese Center for Disease Control and Prevention. The enterovirus D68 strain (EV-D68; GenBank accession number KU844179.1) was a kind gift from Prof. Xiaofang Yu's group at Johns Hopkins University. Influenza A/WSN (WSN) preserved by Tao Wang's group at Tianjin University. Human embryonic kidney 293A (293A) cells, rhabdomyosarcoma (RD) cells and Madin-Darby canine kidney (MDCK) cells were cultured in high-glucose Dulbecco's modified Eagle's medium (DMEM; Invitrogen) supplemented with 10% fetal bovine serum (FBS; Ausbian) and 1% penicillin/streptomycin (Invitrogen) at 37°C and 5% CO_2. Healthy female BALB/C nude mice were purchased from HFK Technology Co., Ltd. (Beijing). The data were shown as mean value ± the standard deviation of independent experiments. In vivo mouse experiments were set as $n \geqslant 3$ for eachgroup. One-way ANOVA and students't test were utilized for statistical analysis. Value of $^*P \leqslant 0.05$, $^{**}P \leqslant 0.01$ were applied to annotate statistical significance.

2. Experimental Procedures

2.1 Animal Ethical Statement

All animal procedures were approved by the Animal Ethics Committee, Tianjin University(TJUE-2024-563) and were conducted in accordance with the Animal Management Rules of the Ministry of Health of the PR China.

2.2 Sensor Preparation

These fluorescent dyes were either developed in our laboratory or donated by companies. Many of the tetrazole-functionalized sensors were newly designed and prepared via organic synthesis. See Supplementary Fig. 3 for the preparation process of the PBET sensor.

2.3 Characterization

The ζ-potentials and size distributions of the viruses were determined with a Zetasizer nanoseries (Malvern instrument). The shape and morphology of the virus samples

were determined via transmission electron microscopy (TEM, Hitachi HT7700). The fluorescence spectra of the virus samples were investigated on an EnSpire multilabel reader. In vitro fluorescence imaging was conducted by placing these PBET-labeled viruses into glass bottom culture dishes. The luminescent signal was recorded with an IVIS Spectrum In Vivo Imaging System (IVIS® Spectrum BL, PerkinElmer).

2.4 Preparation of Virus for Labeling

EV-D68 was prepared in RD cell culture as previously reported. AD5 was prepared in 293A cell culture by incubation with the viral suspension, as previously reported. Once the cells displayed 80% cytopathic effects, the cell culture medium was subjected to three freeze–thaw cycles, followed by centrifugation at 2000 × g for 15 min at 4°C to remove cellular debris. The culture supernatants containing viruses were carefully harvested by ultracentrifugation through SDGC at 110,000 × g for 2 h in an SW41 rotor (Beckman Coulter Inc., Germany). After sucrose was removed, the viruses were sub-packaged and stored at -80°C. SEV viruses were propagated in 10-day-old embryonated eggs for 48 h at 37°C, as previously reported. Subsequently, viruses in allantoic fluid were harvested and purified by SDGC at 110 000 × g in an SW 41 rotor for 2 h at 4°C.

2.5 Virus Labeling Protocol

First, pre-treat the dye molecules by dissolving the PBET sensor in deionized distilled water at room temperature and sonicating it in a water bath for 5 minutes. Immediately centrifuge at 1000×g and collect the supernatant. Next, place pre-chilled purified virus (or an equivalent amount of viral protein) dispersed in various fresh media (e.g., DMEM) into a 10 kDa ultrafiltration tube. Centrifuge at 10,000×g for 5 minutes at 4°C, then redissolve in DMEM to remove cellular components residual from the virus purification process. Mix the obtained PBET solution at a specific concentration with the viral particle solution for labeling. Specifically, mix the working-concentration PBET concentrate with the viral solution at a 20:1 virus/PBET ratio (e.g., add 50 μL PBET solution to 1 mL viral solution). Stir at 4°C in the dark for 30–60 min (extending reaction time does not enhance viral labeling brightness). Transfer the labeled solution to an ultrafiltration tube (10 kDa, Millipore). Centrifuge at 10,000×g for 5 minutes at 4°C. Resuspend the fluorescently labeled complex in fresh sterile DMEM. Repeat washing three times to remove unlabeled and loosely

bound PBET, yielding a stable solution of fluorescently labeled complexes with firmly bound PBET. Typically, aliquot the separated solution to avoid freeze-thaw effects on the virus for subsequent use.

2.6 Modeling of Molecular Docking

The PBET sensor, the VP protein (PDB code: 4WM7) in EV-D68, Capsid protein VP1 (PDB code: 7KFR) in Adeno-Associated Virus and virus F protein in the post-fusion conformation (PDB code: 3MAW) were pre-processed separately via AutoDock Tools 1.6 to generate PDBQT files for molecular docking; PBET and the protein were docked using Vina 1.1.2, and the conformations with the lowest binding energies were selected for analysis. The interactions between proteins and PBET were analyzed using Plip 2021, and three-dimensional interaction maps were drawn using PyMOL 3.0.

2.7 Real-time qPCR

Total RNA was extracted using Trizol (Qiagen). RNAs were reverse transcribed to cDNA via the TransScript First-Strand cDNA Synthesis SuperMix Kit in accordance with the manufacturer's instructions. Quantitative real-time PCR (RT-qPCR) was performed using TransStart Top Green q-PCR SuperMix (TransGen, China) in a 20 μL reaction volume (10 μL TransStart Top Green q-PCR SuperMix, 200 mM forward and reverse primers, 2 μL cDNA template). The following cycling conditions were used: 94°C for 30 s and 40 cycles of 94°C for 5 s and 60°C for 30 s. The reaction was run on a LightCycler 96 Real-Time PCR machine (Roche), and the levels of gene mRNAs were normalized to those of GAPDH mRNA.

2.8 $TCID_{50}$ assay

The infectivity of TPE-labeled viruses was quantified by the 50% tissue culture infective dose ($TCID_{50}$). For the $TCID_{50}$ assay, cells (RD, 293A and MDCK) were cultured in 96-well plates in culture medium until they reached 80–90% confluence. The labeled and nonlabeled viruses were diluted 10^n-fold, ranging from 10^{-4} to 10^{-9} in infection culture media.

2.9 Fluorescence in Vivo Bioimaging

The animal experiments were performed in accordance with the statutory

requirements of the People's Republic of China (GB14925--2010). A solution (100 μL) of the corresponding labeled virus was injected into each mouse via the subcutaneous route or via the tail vein.

2.10 Image Analysis

Imaging data from virus solutions, cultured cells, and animals were processed using ImageJ 1.52p software (NIH). The trajectories of the virus and time courses of fluorescence intensity were reconstructed from the raw images by aligning the coordinates and intensities of the spots representing the virus and corresponding cellular endocytic structures in each frame with Imaging Pro-Plus software (Media Cybernetics). Only the integral trajectories within the focal plane were processed in the quantitative single-virus tracking analysis. The statistical analyses were performed using GraphPad Prism 7 and OriginPro 9.1. Two-tailed Student's t-test, two-way analysis of variance (ANOVA) with Bonferroni's multiple comparisons test and one-way ANOVA with Tukey's multiple comparisons test were performed. The graphs were generated using OriginPro 9.1 (OriginLab).

3. Supplementary Tables

3.1 Supplementary Table 1: Sequence of the proteins used in this study.

Molecule	Sequence
VP1 (EV-D68)<br>PDB ID:<br>pdb_00004wm7	VESIIKTATDTVKSEINAELGVVPSLNAVETGATSNTEPEEAI<br>QTRTVINQHGVSETLVENFLGRAALVSKKSFEYKNHASSSA<br>GTHKNFFKWTINTKSFVQLRRKLELFTYLRFDAEITILTTVA<br>VNGNNDSTYMGLPDLTLQAMFVPTGALTPKEQDSFHWQS<br>GSNASVFFKISDPPARMTIPFMCINSAYSVFYDGFAGFEKNG<br>LYGINPADTIGNLCVRIVNEHQPVGFTVTVRVYMKPKHIKA<br>WAPRPPRTMPYMSIANANYKGRDTAPNTLNAIIGNRASVT<br>TMPHNIVTT
VP1 (AAV-DJ)<br>PDB ID:<br>pdb_00007kfr	MAADGYLPDWLEDTLSEGIRQWWKLKPGPPPPKPAERHK<br>DDSRGLVLPGYKYLGPFNGLDKGEPVNEADAAALEHDKA<br>YDRQLDSGDNPYLKYNHADAEFQERLKEDTSFGGNLGRA<br>VFQAKKRVLEPLGLVEEPVKTAPGKKRPVEHSPAEPDSSSG<br>TGKAGQQPARKRLNFGQTGDADSVPDPQPLGQPPAAPSGL<br>GTNTMASGSGAPMADNNEGADGVGNSSGNWHCDSTWM<br>GDRVITTSTRTWALPTYNNHLYKQISSQSGASNDNHYFGYS<br>TPWGYFDFNRFHCHFSPRDWQRLINNNWGFRPKRLNFKLF<br>NIQVKEVTQNDGTTTIANNLTSTVQVFTDSEYQLPYVLGSA<br>HQGCLPPFPADVFMVPQYGYLTLNNGSQAVGRSSFYCLEY<br>FPSQMLRTGNNFTFSYTFEDVPFHSSYAHSQSLDRLMNPLI<br>DQYLYYLSRTNTPSGTTTQSRLQFSQAGASDIRDQSRNWLP<br>GPCYRQQRVSKTSADNNNSEYSWTGATKYHLNGRDSLVN<br>PGPAMASHKDDEEKFFPQSGVLIFGKQGSEKTNVDIEKVMI<br>TDEEEIRTTNPVATEQYGSVSTNLQGGNTQAATADVNTQG<br>VLPGMVWQDRDVYLQGPIWAKIPHTDGHFHPSPLMGGFG<br>LKHPPPQILIKNTPVPANPSTTFSAAKFASFITQYSTGQVSVE<br>IEWELQKENSKRWNPEIQYTSNYNKSVNVDFTVDTNGVYS<br>EPRPIGTRYLTRNL
F protein (1)<br>(Newcastle<br>disease virus)	MGPRSSTRIPIPLMLTIRIALALSCVHLASSLDGRPLAAAGIV<br>VTGDKAVNIYTSSQTGSIIVKLHPNMPKDKEACAKAPLEAY<br>NRTLTTLLTPLGDSIRRIQESVTTSGGRRQKRFIGAIIGSVAL

PDB ID:<br>pdb_00003maw	GVATAAQITAASALIQANQNAANILRLKESITATIEAVHEVT<br>DGLSQLAVAVGKMQQFVNDQFNNTAQELDCIKITQQVGVE<br>LNLYLTELTTVFGPQITSPALTQLTIQALYNLAGGNMDYLLT<br>KLGVGNNQLSSLIGSGLITGNPILYDSQTQLLGIQVTLPSVG<br>NLNNMRATYLETLSVSTTKGFASALVPKVVTQVGSVIEELD<br>TSYCIETDLDLYCTRIVTFPMSPGIYSCLNGNTSACMYSKTE<br>GALTTPYMTLKGSVIANCKMTTCRCADPPGIISQNYGEAVS<br>LIDRHSCNVLSLDGITLRLSGEFDATYQKNISILDSQVIVTG<br>NLDISTELGNVNNSISNALDKLEESNSKLDKVNVKLTSTSA<br>LITYIALTAISLVCGILSLVLACYLMYKQKAQQKTLLWLGN<br>NTLGQMRATTKM

3.2 Supplementary Table 2: Sequence of the DNA/RNA used in this study.

Molecule	Sequence
EV-D68<br>specific primers	Forward primer: 5′-CACCATACTCACAACTGTGGC-3′;<br>Reverse primer: 5′-AATGAAATGAATCCTGCTCCT-3′.
AD 5<br>specific primers	Forward primer: 5'-GGGTCCGGTTTCTATGCCAA-3'<br>Reverse primer: 5'-CCGTATTCCTCCGGTGATAATG-3'
EV-D68<br>VP-1 RNA	gtggaaagcattattaaaaccgcgaccgataccgtgaaaagcgaaattaacgcggaactg<br>ggcgtggtgccgagcctgaacgcggtggaaaccggcgcgaccagcaacaccgaaccgga<br>agaagcgattcagacccgcaccgtgattaaccagcatggcgtgagcgaaaccctggtggaa<br>aactttctgggccgcgcggcgctggtgagcaaaaaaagctttgaatataaaaaccatgcgagc<br>agcagcgcgggcacccataaaaactttttttaaatggaccattaacaccaaaagctttgtgcagc<br>tgcgccgcaaactggaactgtttacctatctgcgctttgatgcggaaattaccattctgaccacc<br>gtggcggtgaacggcaacaacgatagcacctatatgggcctgccggatctgaccctgcaggc<br>gatgtttgtgccgaccggcgcgctgaccccgaaagaacaggatagctttcattggcagagcg<br>gcagcaacgcgagcgtgtttttttaaaattagcgatccgccggcgcgcatgaccattccgtttatg<br>tgcattaacagcgcgtatagcgtgttttatgatggctttgcgggctttgaaaaaaacggcctgtat<br>ggcattaacccggcggataccattggcaacctgtgcgtgcgcattgtgaacgaacatcagccg<br>gtgggctttaccgtgaccgtgcgcgtgtatatgaaaccgaaacatattaaagcgtgggcgccg<br>cgcccgccgcgcaccatgccgtatatgagcattgcgaacgcgaactataaaggccgcgatac<br>cgcgccgaacaccctgaacgcgattattggcaaccgcgcgagcgtgaccaccatgccgcat<br>aacattgtgaccacc
Adenovirus 5<br>protein V DNA	aagctttaacaaccacgtgcgtacgcttgtggcgcgcgaggaggtggctataggactgatgc<br>atctgtgggactttgtaagcgcgctggagcaaaacccaaatagcaagccgctcatggcgcag<br>ctgttccttatagtgcagcacagcagggacaacgaggcattcagggatgcgctgctaaacata<br>gtagagcccgagggccgctggctgctcgatttgataaacatcctgcagagcatagtggtgca<br>ggagcgcagcttgagcctggctgacaaggtggccgccatcaactattccatgcttagcctggg<br>caagttttacgcccgcaagatataccataccccttacgttcccatagacaaggaggtaaagatc<br>gaggggttctacatgcgcatggcgctgaaggtgcttaccttgagcgacgacctgggcgtttat<br>cgcaacgagcgcatccacaaggccgtgagcgtgagccggcggcgcgagctcagcgaccg<br>cgagctgatgcacagcctgcaaagggccctggctggcacgggcagcggcgatagagaggc<br>cgagtcctactttgacgcgggcgctgacctgcgctgggccccaagccgacgcgccctggag<br>gcagctggggccggacctgggctggcggtggcacccgcgcgcgctggcaacgtcggcgg<br>cgtggaggaatatgacgaggacgatgagtacgagccagaggacggcgagtactaa

4. Supplementary Figures

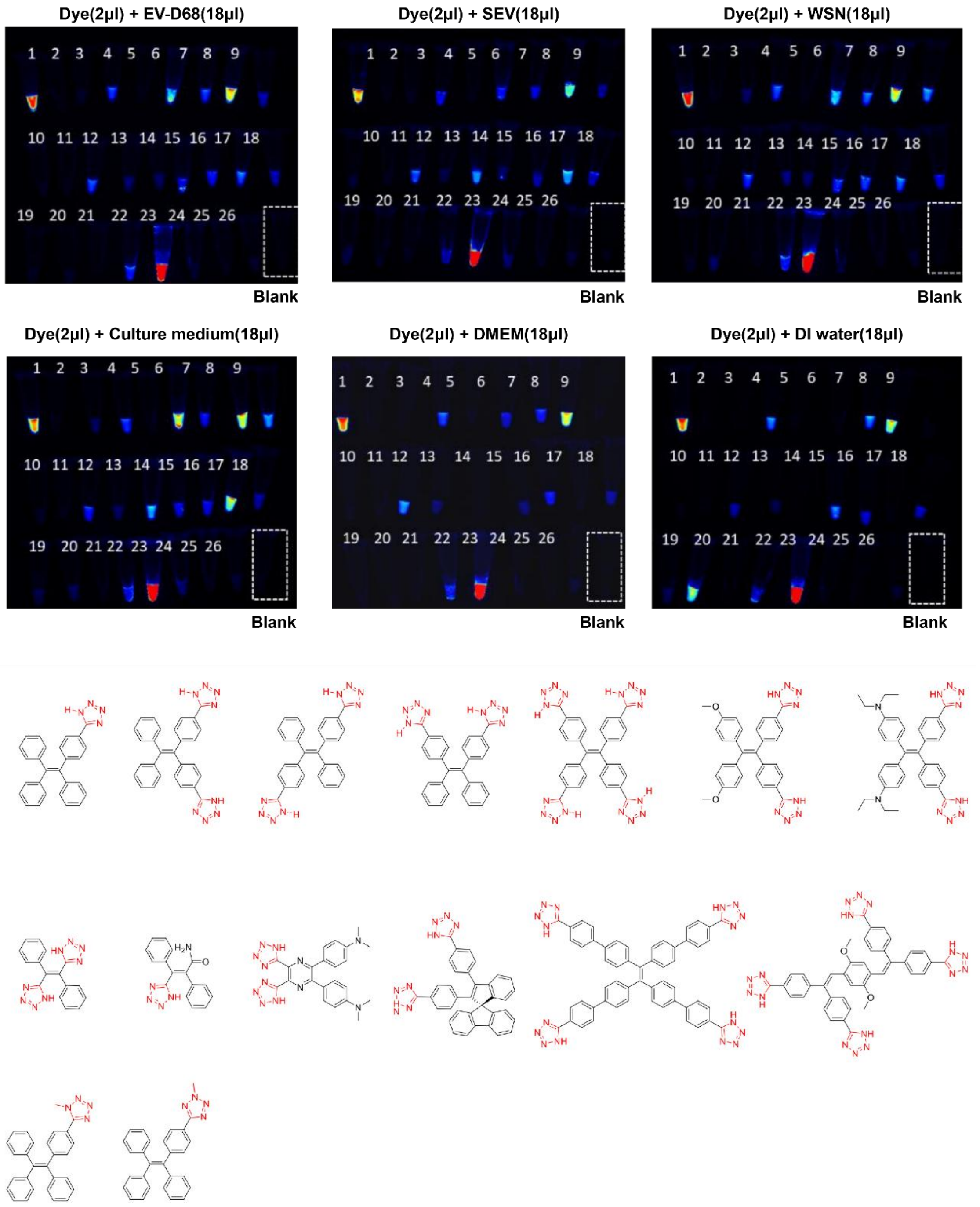

Supplementary Figure 1. The library of tetrazolate-functionalized fluorescent sensor and representative example of screening for fluorescent cores suitable for labeling live viruses.

a. The dye library contains PBET gens, ACQgens (Aggregation-caused quenching luminogens), and some other traditional dyes. [Dye] = 5 μM, [Virus] =10^7 $TCID_{50}$ mL^{-1}. Imaged by in vivo imaging system. **b.** The library of tetrazolate-functionalized fluorescent sensor.

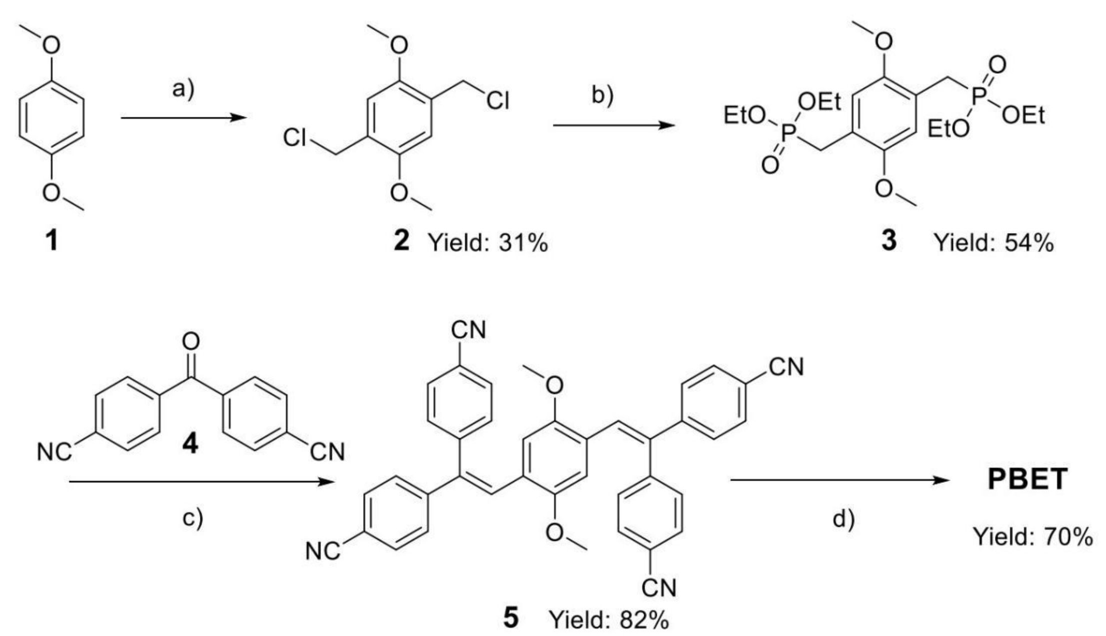

Supplementary Figure 2. Organic synthesis of the PBET dye.

Conditions: a): formaldehyde, paraformaldehyde, HCl, 2 h; **b):** triethylphosphite, reflux, 24 h; **c)** t-BuOK, THF, 4 h; d) NaN_3, $ZnBr_2$, NMP, 8 h.

General information regarding organic synthesis.

All other chemicals and solvents were from Tokyo chemical industry or Sigma-Aldrich. Column chromatography was generally performed on silica gel (200-300 mesh) or neutral alumina and reactions were monitored by thin layer chromatography (TLC) using silica gel GF254 plates with UV light to visualize the course of reaction. Nuclear magnetic resonance (^{1}H NMR and ^{13}C NMR) data were recorded on a 400 MHz spectrometer. High-resolution mass spectra (HRMS) were recorded on an Agilent 1200-6520 Q-TOF mass spectrometer system operating in a MALDI-TOF mode.

a) Synthesis of 1,4-Bis(chloromethyl)-2,5-dimethoxybenzene (Compound 2) (2)

To a solution of 1,4-dimethoxybenzene **(Compound 1)** (10.0 g, 72.3 mmol) in 1,4-dioxane (30 mL), formaldehyde solution (38% in water, 5 mL) and paraformaldehyde (3.0 g, 99.0 mmol) were added. The resulting mixture was stirred at 95°C and concentrated HCl (2 × 5 mL) was added during 30 min intervals.

Heating was continued for 2 h and a further 30 mL of concentrated HCl was added. The resulting mixture was cooled to room temperature to afford a white precipitate, which was collected by filtration and dried under vacuum. The crude product was recrystallized from hot acetone to give product **2** (5.0 g, 29%) as a white precipitate.

^{1}H NMR (300 MHz, $CDCl_3$) δ 6.93 (s, 2H), 4.64 (s, 4H), 3.86 (s, 6H).

b) Synthesis of Compound 3.

To a 100 mL Schlenk tube, **compound 2** (7 g, 29.8 mmol) and triethylphosphite (21 mL, 127 mmol) were added. The tube was purged with nitrogen gas. The reaction mixture was refluxed (at about 154 °C) for 24 h. After that the solution was cooled, the white powder was formed in the tube. The powder was filtered, and then washed with hexanes in a 100 mL flask (50 mL × 3 times, using sonication) . After drying under vacuum, the titled compound was obtained in 54% yield as a white powder (7.0 g).

^{1}H NMR (400 MHz, $CDCl_3$): δ 1.25 (m, 12H), 3.23 (d, 4H), 3.79 (s, 6H), 4.03 (m, 8H), 6.90 (s, 2H).

c) Synthesis of Compound 5.

Under an inert atmosphere, **compound 4** (661 mg, 2.85 mmol), and **compound 3** (500 mg, 1.14 mmol) were dissolved in tetrahydrofuran (25 mL). At room temperature, tetrahydrofuran (6 mL) solution of potassium *tert*-butoxide (383 mg, 3.4 mmol) was added dropwise for 5 minutes, and successively stirred for 4 h. The reaction mixture was charged into water (80 ml), and extracted by DCM (40 mL × 3). The organic layer was washed with water and dried over anhydrous sodium sulfate. The solvent was removed, and the solid was purified by silica gel chromatography using DCM (Rf = 0.8) to give the desired product in 82% yields as yellowish powder.

^{1}H NMR ($CDCl_3$): δ 7.67 (d, 4H, J = 4.0 Hz), 7.61 (d, 4H, J = 4.0 Hz), 7.35 (m, 8H), 7.24 (s, 2H), 6.14 (s, 2H), 3.24 (s, 6H); ^{13}C NMR ($CDCl_3$): δ 151.3, 146.4, 144.8, 139.5, 132.7, 132.3, 131.4, 128.2, 127.0, 125.4, 118.7, 118.3, 112.4, 111.7,

111.5, 55.25 . MS (MALDI-TOF), m/z calcd. For. $C_{40}H_{26}N_4O_2$: 594.21; found 594.20.

d) Synthesis of Compound PBET.

Into a 25 mL flask were added sodium azide (175 mg), zinc bromide (151 mg) and 1 mL water solvent. **Compound 5** (100 mg) was firstly dissolved in 7.5 mL of *N*-Methylpyrrolidone (NMP) and then injected into the above solution. The mixture was stirred overnight for about 8 h at 150°C. The mixture was acidified to pH ~1 with aqueous HCl solution (3M) and was stirred vigorously for another 30 minutes. The organic mixture was extracted with ethyl acetate (20 ml × 2), washed with 3M HCl (50 ml × 2) and concentrated to yield the crude solid. This crude product was added into NaOH solution (0.25 M, 15 ml) and was then stirred vigorously for 1 h. Afterwards, the resulting suspension was filtered to remove the solid. The filtrate was washed with ethylacetate (10 ml × 3) and acidified to pH 1 with 3 M HCl. The tetrazole product precipitated upon stirring, which was again extracted into 20 ml ethyl acetate and the organic layer was separated. The aqueous layer was washed with ethyl acetate (20 ml× 2). The organic layers were combined, concentrated, and dried under vacuum to yield the corresponding **PBET** as a yellow solid (90 mg, 70%).

^{1}H NMR (400 MHz, DMSO-*d6*): δ 8. 18 (d, 4H, J = 4.1 Hz), 8.09 (d, 4H, J = 4.1 Hz), 7.51 (d, 4H, J = 4.2 Hz), 7.41 (d, 4H, J = 4.1 Hz), 7.24 (s, 2H), 6.28 (s, 2H), 3.16 (s, 6H) . ^{13}C NMR (100 MHz, DMSO-*d6*): δ 155.1, 151.1, 144.9, 143.0, 140.7, 131.5, 128.4, 128.3, 127.8, 125.5, 124.8, 124.0, 123.8, 112.7, 100.0, 55.4. MS (MALDI-TOF), m/z calcd. For. $C_{40}H_{30}N_{16}O_2$: 766.27; found 766.28.

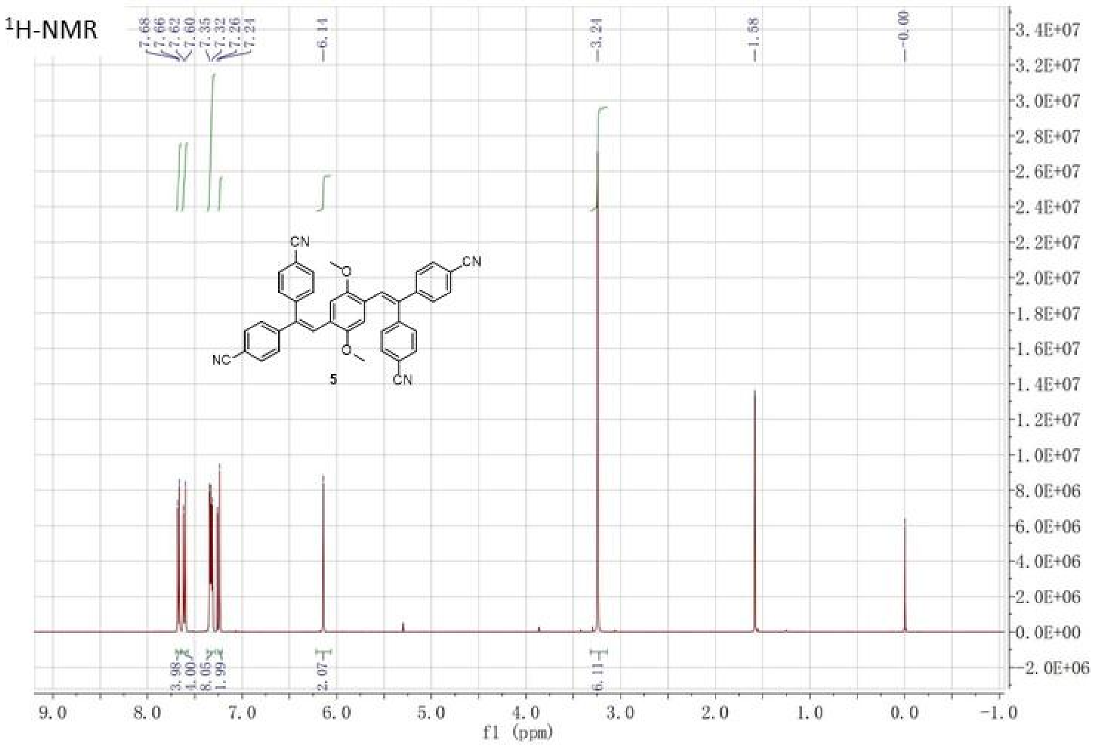

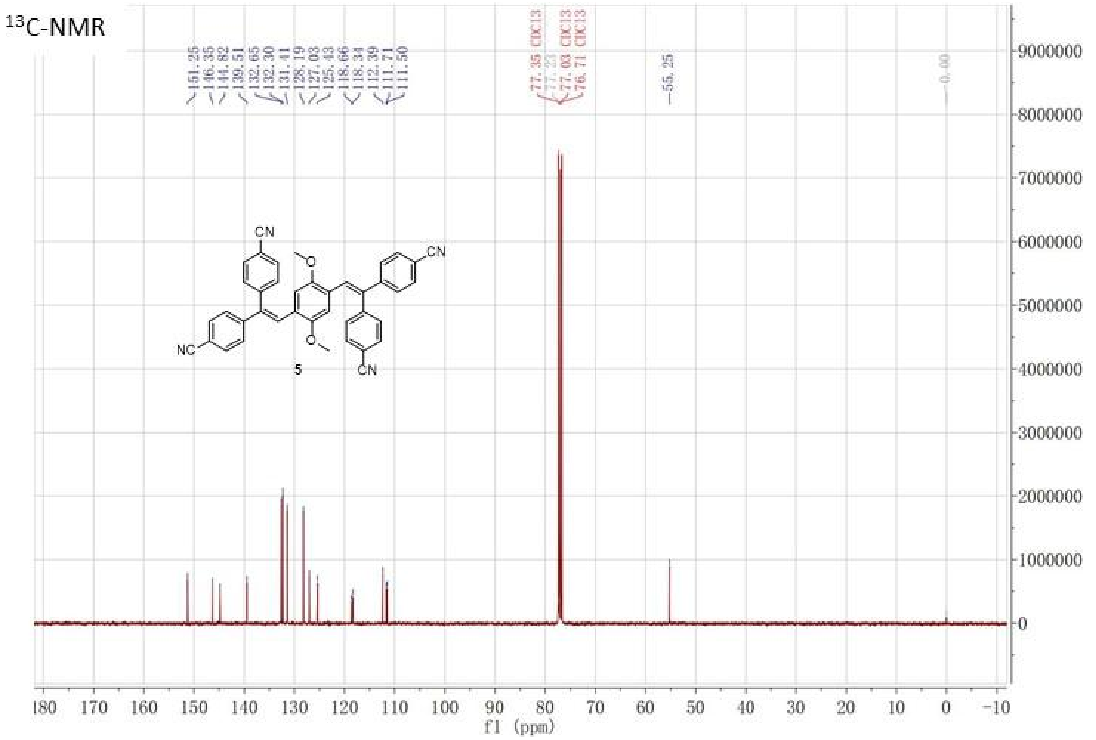

Supplementary Figure 3. NMR characterization of compound 5.

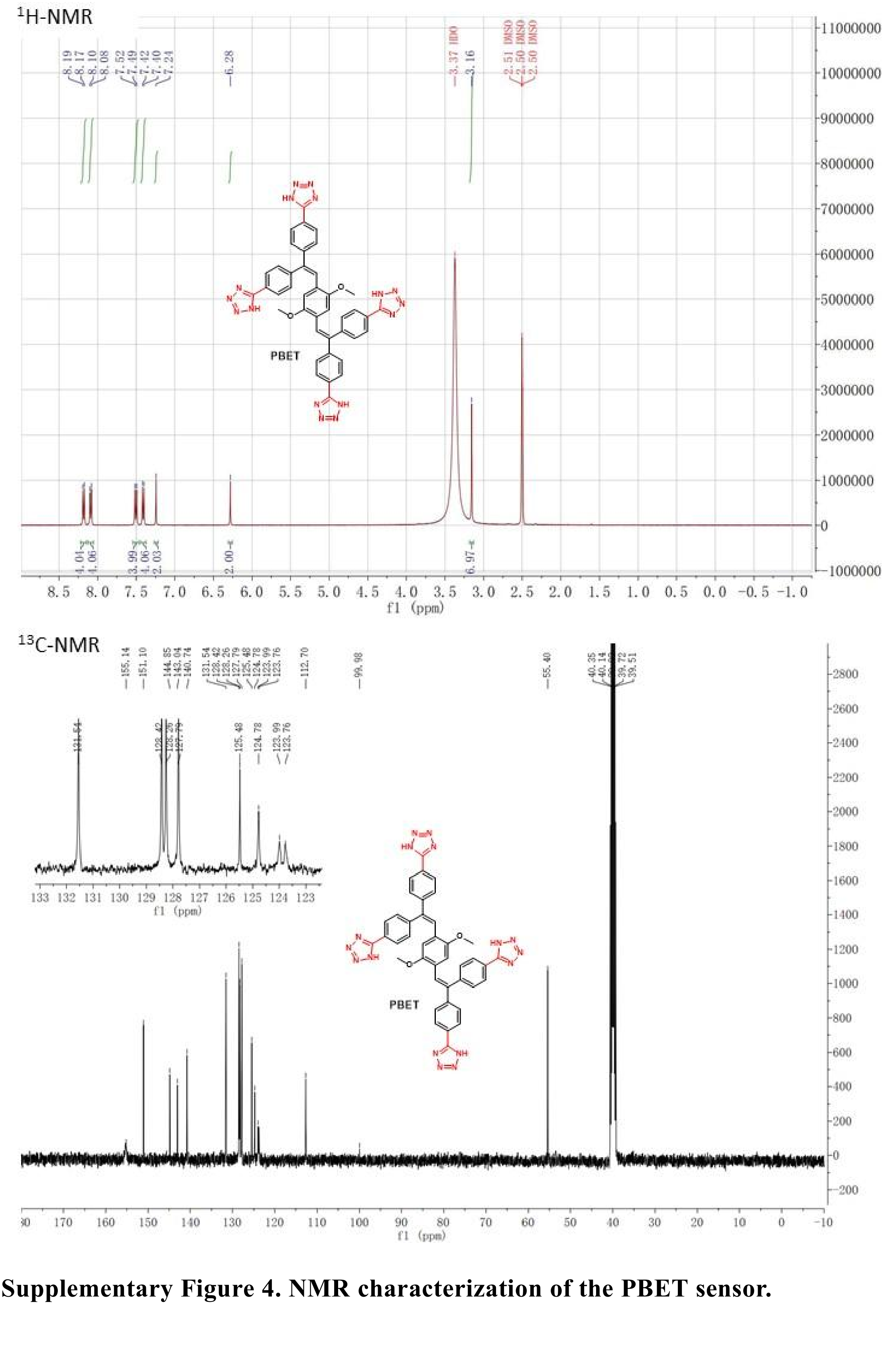

Supplementary Figure 4. NMR characterization of the PBET sensor.

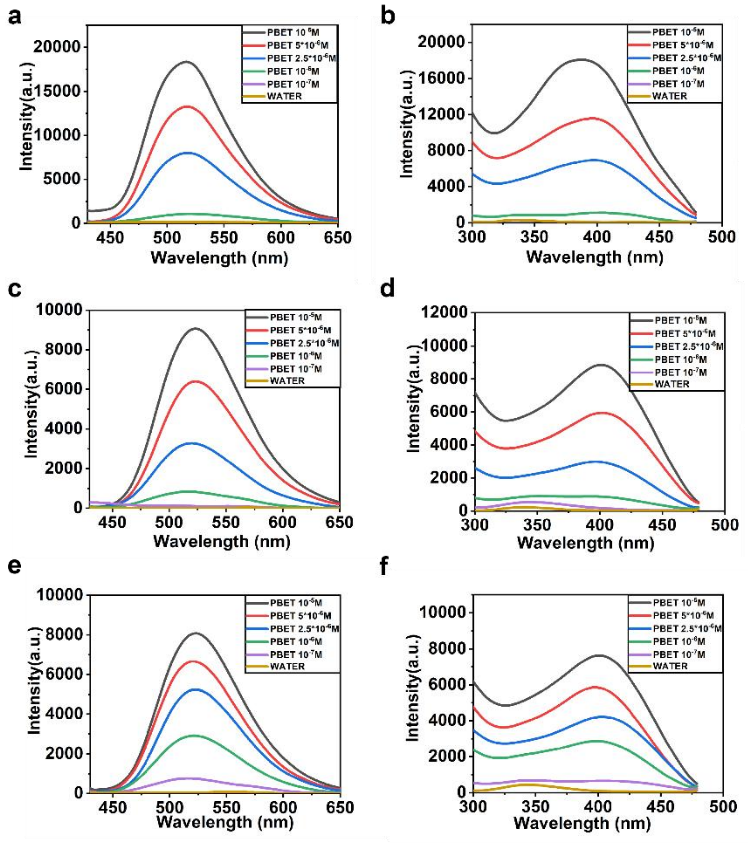

Supplementary Figure 5. Fluorescent titration study of the different concentrations of virus and the PBET dye.

a-f. Emission spectra of PBET binding in water environments: AD-5 **(a)**, EV-D68 **(c)**, and SEV **(e)**. d. Excitation spectra of PBET binding in water environments: AD-5 **(b)**, EV-D68 **(d)**, and SEV **(f)**. [PBET] from 0 to 10^{-5}M + [Virus] (10^{7} TCID50 mL^{-1}) .

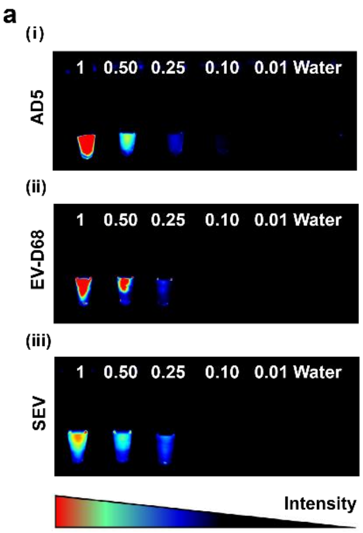

Supplementary Figure 6. Fluorescent study of the different types of PBET@virus.

a. Fluorescence images of PBET dye (5μM) under different virus types and titers. [PBET] 5μM. [Virus] (10^7 TCID50 mL^{-1}) .

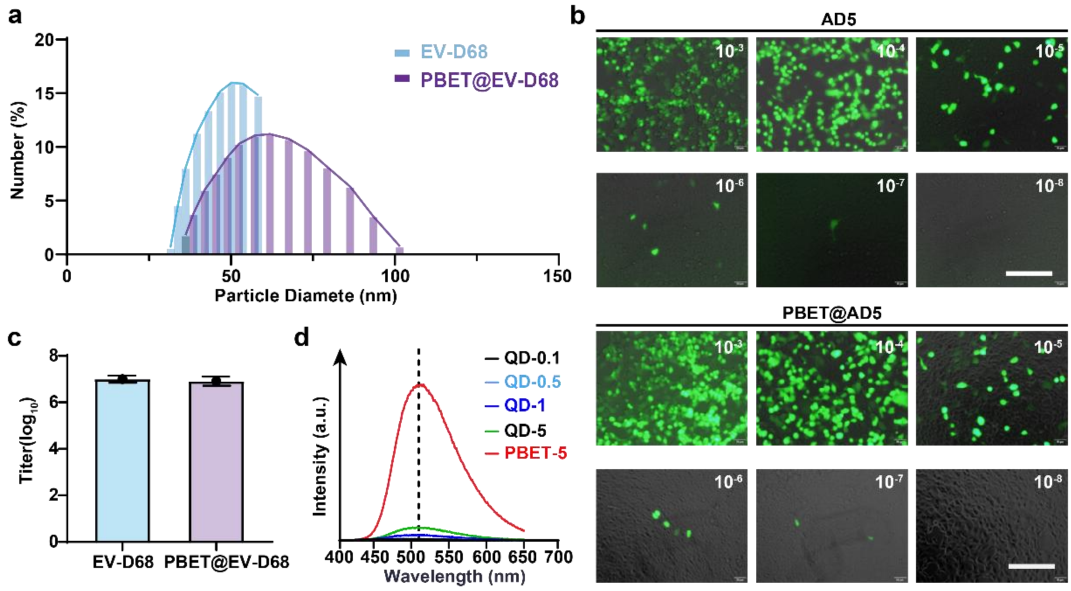

Supplementary Figure 7. Effect of PBET fluorescent sensor on the physicochemical properties of viruses.

a. DLS measurement of EV-D68 before and after labeling with PBET. **b.** Quantification of transfection by loading EGFP plasmid AD5 virus with PBET before and after mixing with PBET in the same cell incubation; fluorescence pictures show green fluorescence expression after transfection. Scale bar: 200μm. **c.** Titer test of PBET mixed with EV-D68. **d.** Fluorescence curves of QD-labeled viruses or PBET fluorescently labeled complexes at different concentration gradients. [PBET] = 5μM.

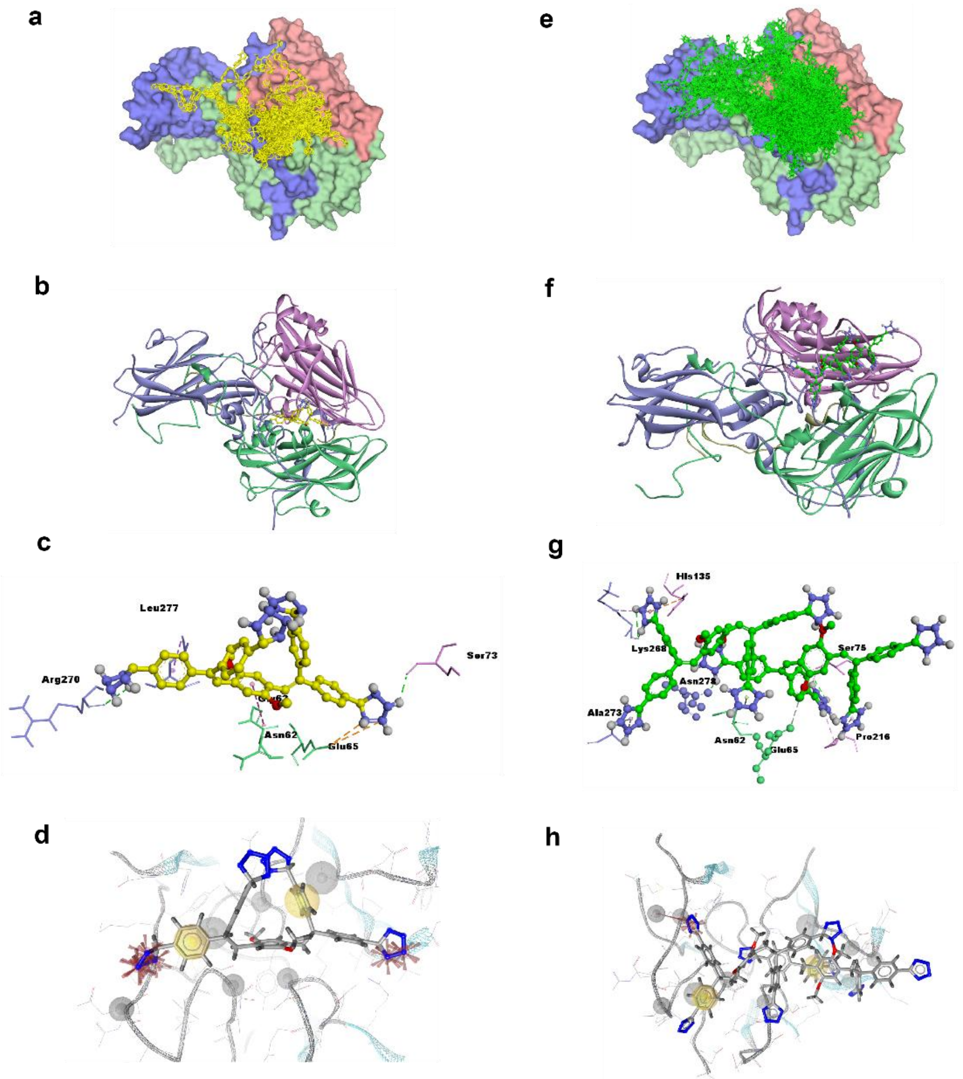

Supplementary Figure 8. Molecular Docking Model of the PBET-EV-D68 Surface Capsid Protein Complex.

a. Top 50 docking binding sites of PBET monomer with VP complex. **b.** Example of PBET monomer docking binding site with VP complex. **c.** Molecular interactions of PBET monomer within VP complex docking pocket. **d.** Ligand pharmacophore model of PBET monomer. **e.** Top 50 docking binding sites of PBET dimer in VP complex. **f.** Example docking binding site of PBET dimer in VP complex. **g.** Molecular interactions of PBET dimer within VP complex docking pocket. **h.** Ligand pharmacophore model of PBET dimer.

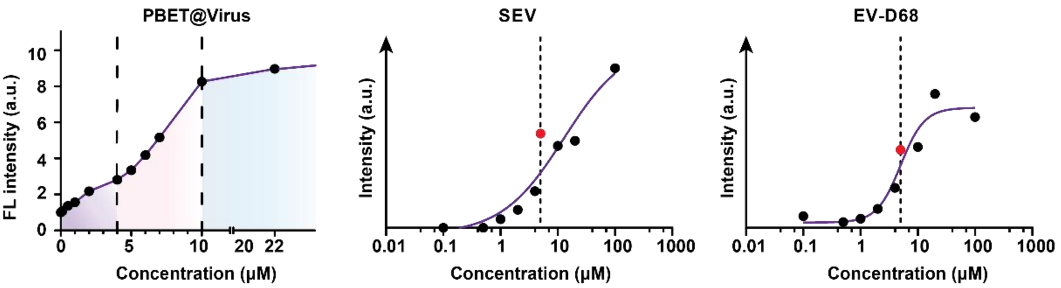

Supplementary Figure 9. PBET Concentration Gradient Fluorescence Emission Peak Regression Fitting.

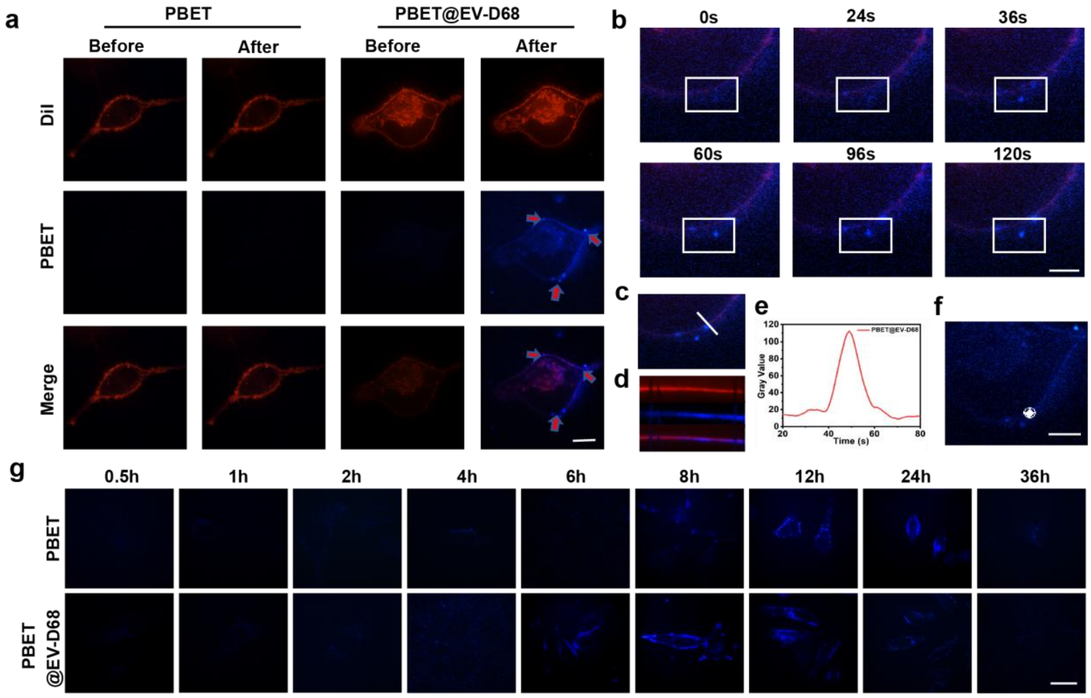

Supplementary Figure 10. Fluorescence imaging of PBET-labeled live EV-D68 virus in RD cells.

a. Confocal fluorescence images of cell which are surrounded by EV-D68 viruses. Red: from cell membrane specific Dil dye. Blue: from PBET-labeled EV-D68 viruses. Scalebar: 25 μm. **b.** Snapshots of the entry of a PBET-labeled virus shown in a Video, recorded by via a Confocal fluorescence microscopy. Scalebar: 5 μm. **c,d.** Kymograph of the binding site of the virus. **e.** the corresponding fluorescence intensity curve (Middle curve). Scalebar: 1 μm. The distance between two neighboring pixels is about 12.5 nm. **f.** Trajectory of viral diffusion from sequential (**b**) images. **g.** Long-term tracking of PBET-labeled EV-D68 viruses in cells. Scale bar: 25 μm.

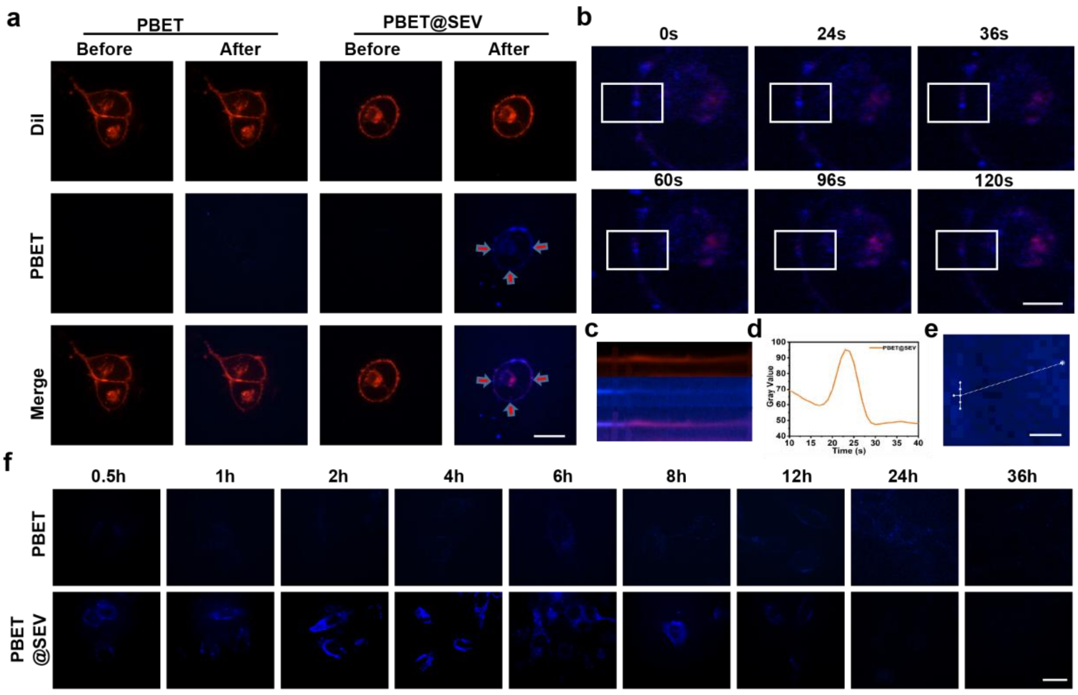

Supplementary Figure 11. Fluorescence imaging of PBET-labeled live SEV virus in RD cells.

a. Confocal fluorescence images of cell which are surrounded by SEV viruses. Red: from cell membrane specific Dil dye. Blue: from PBET-labeled SEV viruses. Scalebar: 25 μm. **b.**Snapshots of the entry of a PBET-labeled virus shown in a Video, recorded by via a Confocal fluorescence microscopy. Scalebar: 5 μm. **c.** Kymograph of the binding site of the virus. **d.** the corresponding fluorescence intensity curve (Middle curve). Scalebar: 1 μm. The distance between two neighboring pixels is about 12.5 nm. **e.** Trajectory of viral diffusion from sequential **(b)** images. **f.** Long-term tracking of PBET-labeled SEV viruses in cells. Scale bar: 25 μm.

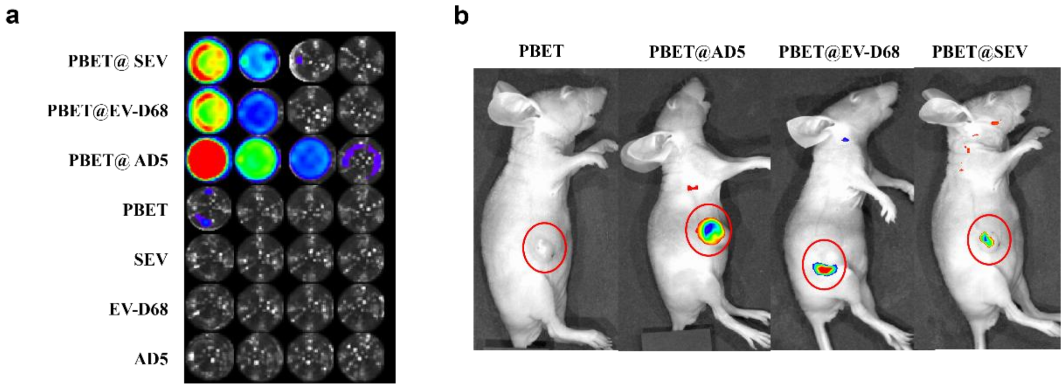

Supplementary Figure 12. In Vivo Bioimaging Study Evaluates PBET Animal Model Visualization Potential.

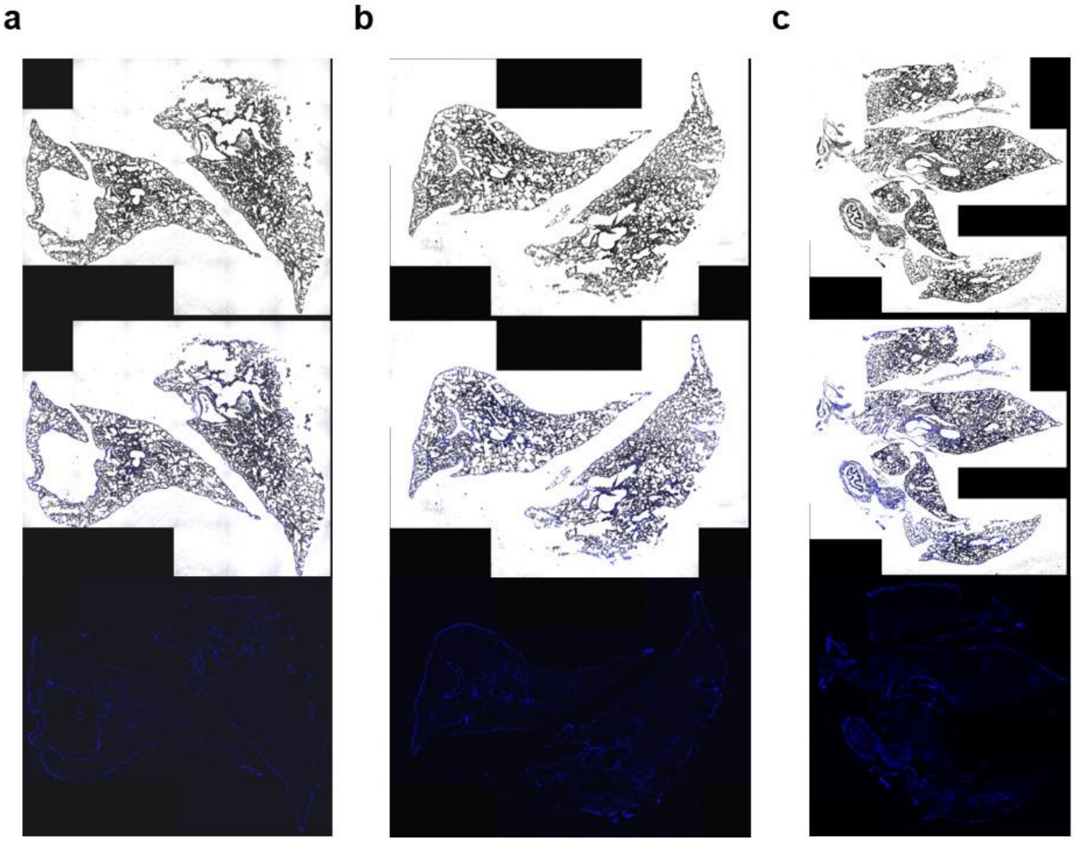

Supplementary Figure 13. Tissue fluorescence scanning of a lung infection model removed in vivo, with labeled virus particles indicated in blue.

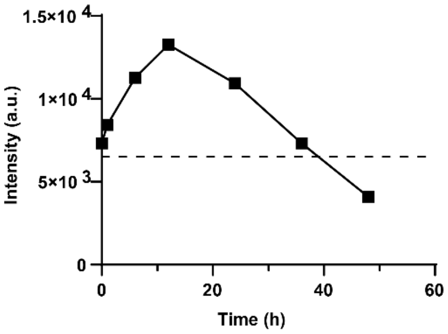

Supplementary Figure 14. Fluorescence half-life of PBET@EV-D68 in serum.

5. References

1. Ludwig K. 2003. The 3D structure of the fusion primed sendai F-protein determined by electron cryomicroscopy. *The EMBO Journal* 22:3761–3771.

2. Jeon S, Park S, Nam J, Kang Y, Kim J-M. 2016. Creating patterned conjugated polymer images using water-compatible reactive inkjet printing. *ACS Appl Mater Interfaces* 8:1813–1818.
